# Closing the genome of unculturable cable bacteria using a combined metagenomic assembly of long and short sequencing reads

**DOI:** 10.1099/mgen.0.001197

**Published:** 2024-02-20

**Authors:** Anwar Hiralal, Jeanine S. Geelhoed, Silvia Hidalgo-Martinez, Bent Smets, Jesper R. van Dijk, Filip J. R. Meysman

**Affiliations:** ^1^​ Geobiology Research Group, University of Antwerp, Antwerp, Belgium; ^2^​ Department of Biotechnology, Delft University of Technology, Delft, Netherlands

**Keywords:** genome closure, cable bacteria, metagenomics, hybrid sequencing, clonal enrichment, *Candidatus *Electrothrix scaldis

## Abstract

Many environmentally relevant micro-organisms cannot be cultured, and even with the latest metagenomic approaches, achieving complete genomes for specific target organisms of interest remains a challenge. Cable bacteria provide a prominent example of a microbial ecosystem engineer that is currently unculturable. They occur in low abundance in natural sediments, but due to their capability for long-distance electron transport, they exert a disproportionately large impact on the biogeochemistry of their environment. Current available genomes of marine cable bacteria are highly fragmented and incomplete, hampering the elucidation of their unique electrogenic physiology. Here, we present a metagenomic pipeline that combines Nanopore long-read and Illumina short-read shotgun sequencing. Starting from a clonal enrichment of a cable bacterium, we recovered a circular metagenome-assembled genome (5.09 Mbp in size), which represents a novel cable bacterium species with the proposed name *Candidatus* Electrothrix scaldis. The closed genome contains 1109 novel identified genes, including key metabolic enzymes not previously described in incomplete genomes of cable bacteria. We examined in detail the factors leading to genome closure. Foremost, native, non-amplified long reads are crucial to resolve the many repetitive regions within the genome of cable bacteria, and by analysing the whole metagenomic assembly, we found that low strain diversity is key for achieving genome closure. The insights and approaches presented here could help achieve genome closure for other keystone micro-organisms present in complex environmental samples at low abundance.

## Data Summary

Genomic data for this study were deposited at the National Center for Biotechnology Information (NCBI) under BioProject ID PRJNA1030987. All amplicon sequences can be found at the European Nucleotide Archive (ENA) under BioProject ID PRJEB67707.

**Impact Statement**

This study presents a novel approach for obtaining closed genomes of unculturable bacteria, using the enigmatic cable bacteria as a case study. The approach involves the development of a clonal enrichment culture and metagenomic hybrid sequencing, and we present a systematic investigation into the factors that enable genome closure. As a result, the first closed genome of the genus *Candidatus* Electrothrix was obtained (of the novel species *Ca*. Electrothrix scaldis), which allowed us to unveil key genes previously missed in incomplete assemblies.

## Introduction

As of September 2023, 1.6 million bacterial assemblies have been submitted to the database of the National Center for Biotechnology Information (NCBI), but only 2.5 % of these assemblies were categorized as ‘complete genomes’. Yet, to fully understand the metabolic capacity of any micro-organism, complete (i.e. closed and high-quality) genomes are of prime importance. The main obstacle in attaining closed genomes is repetitive sequences, which particularly pose problems when short-read sequencing platforms are exclusively used [1]. Because of repetition, shorter reads cannot be anchored to unique genomic regions during genomic assembly, causing fragmentation into multiple contigs [2]. Genome fragmentation poses difficulties for downstream analyses, such as comparative genomics, core genome analysis and investigation of gene synteny, which all rely on genomes with high contiguity [3].

Recently, the emergence of long-read sequencing platforms such as Oxford Nanopore Technologies (ONT) and Pacific Biosciences (PacBio) have markedly facilitated the closure of microbial genomes [4, 5]. The long reads produced can span repetitive elements and thus anchor to unique genomic regions, enabling closed genomes to be produced in a single sequencing run [6, 7]. Because long-read sequencing technologies tend to be less accurate than short reads, a combination of short- and long-read sequencing platforms has become the preferred approach to obtain closed and highly accurate genomes [8, 9]. For micro-organisms that can be grown in axenic cultures, this ‘hybrid sequencing’ method has now become the gold standard for generating complete genomes (e.g. [10–14]).

Axenic cultures provide large quantities of biomass (and thus DNA) of a single microbial strain, which facilitates genome assembly by having a high sequencing coverage depth for a single genome. However, the vast majority of micro-organisms are unculturable, and hence axenic cultures are not available [15–17]. Additionally, target organisms are typically present at low abundance in environmental samples, and only represent a small fraction of the total DNA pool [18]. Single-cell genomics and metagenomics provide suitable solutions to this problem [19, 20], but when performed with short-read sequencing platforms, the resulting genomes often remain incomplete. Recently, the combined metagenomic assembly of long and short sequencing reads has shown great promise for generating closed genomes for unculturable micro-organisms [21]. However, successful application appears to be limited to micro-organisms that have a relatively high abundance and low strain diversity compared to the other members of the same microbiome [21, 22].

As such, an important remaining challenge is the generation of closed genomes for community members that are not highly abundant and/or display high strain diversity but do play a vital role in ecosystem functioning. An example of such ‘microbial ecosystem engineers’ are cable bacteria, centimetre-long filamentous bacteria that are capable of performing long-distance electron transfer [23, 24]. Cable bacteria can be found globally in freshwater and saltwater sediments [25–27], and when active, their electrogenic metabolism exerts a large impact on the geochemistry of the sediments, as it strongly influences the cycling of sulfur, iron and phosphorus [28, 29]. Currently, all cable bacteria are classified into two candidate genera: *Candidatus* Electronema, typically found in freshwater environments, and *Candidatus* Electrothrix, typically found in saltwater environments [30, 31].

While cable bacteria have a strong impact on the biogeochemical cycling of natural sediments [28, 29, 32–34], they are generally present in relatively low abundances (0.01–1 % [35, 36]) and only occasionally reach higher densities (4.5 % [35, 37]). Moreover, multiple strains of closely related cable bacteria are typically found within the same sediment environment [37, 38], and the microbial community of natural sediment environments is known to be highly diverse [39, 40]. This particular combination of factors makes the metagenomic assembly of cable bacteria genomes challenging. Only recently, the first closed genomes of the genus *Ca*. Electronema were obtained through a hybrid sequencing approach [41]. Yet, for the genus *Ca*. Electrothrix that dominates marine environments genome closure has not yet been achieved.

Here, we present a novel approach to generate closed and high-quality genomes for the unculturable cable bacteria, which is based on the generation of a clonal enrichment and the combined application of Nanopore long-read and Illumina short-read shotgun sequencing. This approach results in a closed genome, which represents a novel species within the genus *Ca*. Electrothrix. Following that, we systematically investigated the factors that resulted in genome closure. Finally, we provide new insights into the physiology of cable bacteria that are unveiled by the acquired closed genome.

## Methods

### Clonal enrichment culturing

Two separate cable bacteria enrichment cultures were prepared for this study. A ‘natural enrichment culture’ that typically contains multiple cable bacterium strains [26], as well as a so-called ‘clonal enrichment culture’ that is selectively prepared and dominated by a single cable bacterium strain [42]. Both enrichment cultures were prepared from natural sediment collected from a creek bed at Rattekaai salt marsh in the Netherlands (51.4391°N, 4.1697°E). Earlier studies at this location have confirmed the presence of cable bacteria *in situ* [26]. Before incubation, sediment was sieved (<1.1 mm) to remove large debris and fauna, and homogenized.

To obtain the natural enrichment culture, sieved and homogenized sediment was filled into polycarbonate core liners (inner diameter of 4 cm), and submerged in a container with continuously aerated artificial seawater (salinity 30; Instant Ocean, Blacksburg, USA). Cores were incubated at room temperature in the dark for several weeks until the metabolic activity of cable bacteria became apparent from the distinct discolouration of the sediment and the appearance of a characteristic geochemical fingerprint, as recorded by microsensor depth profiling [24, 26].

For the clonal enrichment culture, sediment was semi-sterilized by autoclaving in a sealed bottle under a N_2_ atmosphere (~0.2 l sediment, 25 min at 121 °C). After cooling, the semi-sterile sediment was filled into glass cores (inner diameter of 13 mm). A few days later, after an oxic zone of ~1 mm had developed, the sediment was inoculated with a single cable bacterium filament. To achieve this, we used micromanipulation with custom-developed glass hooks to retrieve an individual cable bacteria filament from a natural enrichment culture with an active cable bacteria population. Cores were capped, incubated at room temperature in the dark for several weeks and exposed to air instead of overlying seawater. The active clonal enrichment culture was maintained in the laboratory by regularly transferring a small amount of the clonal enrichment culture to freshly prepared semi-sterilized sediment. The fifth generation of the clonal culture was used for subsequent DNA extraction and sequencing.

### Fluorescence *in situ* hybridization (FISH), scanning electron microscopy (SEM) and Raman microscopy

Individual filaments were harvested from the fifth generation of the clonal enrichment culture using small, custom-developed glass hooks under a stereomicroscope [42]. Filaments were washed 6–10 times in droplets (~20 µl) of MilliQ water (mQw) to remove sediment particles and salts. Different microscopic imaging techniques were used to verify that the observed filaments were indeed cable bacteria.

To perform FISH, picked filaments were fixed in a 1 : 1 phosphate-buffered saline (PBS)/ethanol mixture for 5 s and subsequently rinsed in mQ. The filaments were then transferred onto a 13 mm diameter polycarbonate membrane filter (pore size 0.2 µm, Merck Millipore) and embedded in 1 % low-melting-point agarose. Hybridization was performed according to previously described protocols [43, 44], with Cy5-labelled probe DSB706 that targets *Desulfobulbaceae* [45, 46]. Samples were counterstained with 1 µg ml^−1^ 4′,6-diamidino-2-phenylindole (DAPI) in 8 : 2 Citifluor/Vectashield and analysed using an epifluorescence Zeiss Axioplan 2 microscope with a Cool LED pE-300 light source.

To perform SEM, picked filaments were deposited on a 13 mm diameter polycarbonate membrane filter (0.2 µm pore size, Merck Millipore). After drying and gold coating (Agar Sputter Coater), SEM images were obtained with a Phenom ProX scanning electron microscope (Phenom-World B.V., the Netherlands) using a backscattered electron detector at an acceleration voltage of 10 kV.

To perform Raman microscopy, filaments were transferred to a droplet (~2 µl) of mQw on a piece of gold-coated silicon wafer (50 nm gold layer, Platypus Technologies) and left to dry. Raman spectra were collected using a Renishaw inVia Qontor confocal Raman microscope with a 532 nm excitation laser. The excitation laser was focused using a 100× microscopy objective (NA 0.9) and delivered ~1.25 mW of power on the sample. The optical components of the microscope included a 65 µm confocal aperture and 1800 l mm^−1^ grating. Raman scattered light was captured with a thermoelectrically cooled (−70 °C) charged coupled device (CCD) detector. The Raman system was calibrated using the response of an internal Si standard. Raman spectra were acquired over a range from 100 to 3200 cm^−1^, using an acquisition time of 30 s. The laser spot was focused on the centre part of individual cells, allowing us to collect spectra from multiple cells within different cable bacteria filaments. Spectra were baseline-corrected in LabSpec6 (version 6.6.1.11; Horiba) by fitting a polynomial to the Raman signal. Finally, all Raman spectra were averaged to obtain a final spectrum.

### Sediment sampling and DNA extraction

DNA was extracted from specific layers in the enrichment cultures. To this end, individual sediment cores were subsectioned at specific depth resolutions (0.2 cm for natural enrichment cultures, 0.3 cm for the clonal enrichment culture). DNA extraction was performed as previously described [47] using 0.5 g wet sediment for the natural enrichment cultures and 0.25 g wet sediment for the clonal enrichment culture.

DNA was quantified using Qubit 3.0 and the Qubit dsDNA HS assay kit (Life Technologies, Thermo Fisher Scientific). To obtain sufficient DNA for Nanopore and Illumina sequencing from the clonal enrichment culture, 0.3–0.6 cm and 0.6–0.9 cm sections were pooled (total DNA quantity ~5 µg). The fragment length of extracted DNA was checked using a fragment analyser (Agilent 5300), showing an average size of 19 kbp.

### Amplicon sequencing and analysis

To analyse the microbial community of the enriched sediments, the V3–V4 region of the 16S rRNA gene was amplified using primers 341F (5′-CCTACGGGNGGCWGCAG-3′) and 785 R (5′-GACTACHVGGGTATCTAATCC-3′) with Illumina adapter sequences as overhang [48]. PCR was performed using Phusion High-Fidelity DNA polymerase (New England Biolabs), with the following cycling parameters: initial denaturation at 98 °C for 1 min, followed by 27 cycles of denaturation at 98 °C for 30 s, annealing at 57 °C for 30 s and an extension at 72 °C for 30 s. The final extension was performed for 5 min at 72 °C. Library preparation and sequencing (Illumina MiSeq 2×300 bp) were performed by Eurofins Genomics, Konstanz, Germany.

Amplicon sequence variants (ASVs) were analysed using the dada2 pipeline in R [49]. Reads were filtered and merged using the default parameters of the pipeline, except the following parameters during the merging steps: trimLeft=c(17,21), truncLen=c(272,216) and truncQ=4. Singletons and chimaeras were removed. Taxonomy was assigned using the Silva small subunit rRNA database v138.1 [50]. Alpha diversity indices were calculated using the phyloseq package for R [51].

### Single-filament DNA sequencing

Individual filaments were collected from the clonal enrichment culture using micromanipulation with custom-made glass hooks [42]. Cells were lysed at 95 °C for 15 min. DNA was amplified using multiple displacement amplification (MDA) with the REPLI-g Single Cell kit (Qiagen), according to the manufacturer’s instructions. Amplified DNA was used to perform Oxford Nanopore MinION sequencing and Illumina HiSeq 2500 sequencing. For Nanopore, library preparation was performed using the SQK-RBK004 rapid barcoding kit (Oxford Nanopore Technologies, ONT), according to the manufacturer’s instruction (DNA quantity 150 fmol). Several filaments were sequenced simultaneously, but only one barcode was used for this study. The library was run on a FLO-MIN106 flowcell (9.4 chemistry, ONT) for 48 h. Illumina HiSeq 2500 sequencing and library preparation was performed by Eurofins Genomics, Konstanz, Germany.

### Metagenomic DNA sequencing

DNA extracted from the clonal enrichment culture was sequenced using Nanopore and Illumina sequencing by the Neuromics support facility (Flanders Institute for Biotechnology – University of Antwerp). Nanopore library preparation was performed using the SQK-LSK109 ligation sequencing kit (ONT), according to the manufacturer’s instruction (DNA quantity 150 fmol). As three other experiments were also to be sequenced simultaneously (not applicable for this study), the EXP-NBD114 native barcoding kit (ONT) was used to barcode and pool the samples at equimolar concentrations. The library was run on a FLO-MIN106 MinION flowcell (9.4 chemistry, ONT) for 80 h, with half of the total library loaded initially and the other half added after 48 h. An Illumina MiSeq library was prepared using the Nextera XT DNA Library Preparation kit (Illumina) and sequenced using a Illumina Miseq system with the MiSeq reagent V3 kit flow cell.

### Basecalling and read processing

Illumina data was quality-checked using FastQC v0.11.750 (https://github.com/s-andrews/FastQC}) and MultiQC v1.751 [52]. Cutadapt v1.1652 was used to trim reads from the 3′ end with a quality cutoff of *q*=20, removing sequencing adapters, reads < 100 bp and ambiguous bases (N) from the start and end using the parameters -m 100 -q 20 --max-n=COUNT 0 --trim-n [53].

Raw Nanopore fast5 data were basecalled using Guppy v2.2.3 for MinION and the dna_r9.4.1_450bps_hac.cfg model from ONT; 9.11 Gbp of data from this sample were obtained after basecalling [54]. The data were explored using MinionQC v1.4.049 [55]. Samples were demultiplexed, and barcodes and adapter sequences trimmed using qcat v1.0.1 (https://github.com/nanoporetech/qcat), using the flags -b -k NBD103/NBD104 --trim --detect-middle. Following demultiplexing, the Nanopore reads were further processed using Filtlong v0.2.0 (https://github.com/rrwick/Filtlong) to remove reads <4000 bp using --min_length 4000 and to remove low-quality reads with <80 % base call accuracy using --min_mean_q 80. Porechop v0.2.3 (https://github.com/rrwick/Porechop) was used to check the reads for residual barcodes and adapters using default settings and the flag --min_split_read_size 4000.

### Short-read metagenome assembly and binning

Illumina reads were assembled using the metaspades pipeline of SPAdes v3.14.1 with default parameters [56]. Contigs shorter than 1000 bp were discarded. Contigs were mapped to the closed, polished genome (see below) using minimap2 v2.1657 with the ‘sr’ preset [57]. Partially aligned and unaligned contigs were discarded using Samtools v1.958 [58].

### Long-read metagenome assembly and polishing

The quality-processed Nanopore reads were assembled using Flye v1.2 with the --meta parameter [59, 60]. Contigs< 1000 bp were removed using seqtk v1.3-r106 (https://github.com/lh3/seqtk). Circularity was assessed by manual inspection of mapped reads that overlapped at the start and end of contigs in Tablet v1.21.02.08 [61].

The initial four rounds of Nanopore-based assembly polishing were performed using Racon v1.3.3 with the argument ‘--include-unpolished’ for initial correction [62]. These were followed by two rounds of medaka v0.6.5 (https://github.com/nanoporetech/medaka) polishing, using the medaka consensus command. A final round of error correction was performed using the quality-processed MiSeq reads (derived from the sediment sample) using Racon v1.3.3 with standard parameters. Samtools v1.958 and minimap2 v2.1657 were used as a dependence for all polishing steps [57, 58]

### MAG binning and dereplication

MaxBin v2.2.760 and MetaBAT2 v2.12.159 [63, 64] were used for automated binning. DASTool v1.1.164, with --search_engine diamond, was used for dereplication [65]. Each MAG was evaluated for genome completeness using CheckM v1.2.2 with the preset --lineage_wf [66]. 16S rRNA genes were identified with barrnap v0.9 (https://github.com/tseemann/barrnap), and then classified with MOTHUR v2.7.14 ‘--classify.seqs’ against the silva v138.1 database [50, 67].

### Relative abundance, strain heterogeneity and contiguity

To determine the relative abundance of each MAG, we mapped the Illumina reads to the whole metagenomic assembly using minimap2 v2.1657 and BWA v.0.7.17 [68]. After, CoverM v0.3.2 (https://github.com/wwood/CoverM) was used with the following arguments: coverm genome -m relative abundance --min-read-aligned-percent 0.9 --min-read-percent-identity 0.95 --min-covered-fraction 0. Strict parameter values were set to exclude false-positive mappings. To determine the strain heterogeneity of each MAG, polymorphic rates were calculated using CMSeq v1.0.2 [69]. The polymut.py and poly.py scripts were used with the following parameters: -mincov 10 --minqual 30 --dominant_frq_thrsh 0.8. To determine the contiguity of each MAG, the contig N50 was taken and divided by the total size.

### Annotation, taxonomy and functional analysis

GTDB-Tk v1.6.0 was used to classify genomes based on their placement in the reference tree using ‘classify_wf’ [70]. Additionally, ‘de_novo_wf’ was used to infer a *de novo* tree using all MAGs.

Gene calling of the cable bacterium CMAG was performed with prodigal v2.6.3 [71] in Prokka v1.14 --kingdom ‘Bacteria’ [72]. Insertion sequences were detected using ISEScan v1.7.2.3 [73]. Repeats were using MUMmer4 with nucmer --maxmatch -l 100 settings in a self-versus-self search [74].

Each available cable bacteria genome [30, 41, 75] was evaluated for genome completeness using CheckM v1.2.2 with the preset --lineage_wf [66]. To determine the ‘single genes’ of *Ca*. Electrothrix scaldis, genomes of the genus *Ca*. Electrothrix were used as input in the GET_HOMOLOGUES v3.6.1 pangenome analysis pipeline [76]. Using GET_HOMOLOGUES, a consensus of protein clusters was created using the BDBH, COGtriangle and OMCL algorithms (with default parameters), and these clusters were used as a basis to identify the protein sequences unique to *Ca*. E. scaldis. Functionality was assigned using EggNOG-mapper v2.1 and used to assign cluster of orthologous group (COG) functionalities [77].

To identify sulfur metabolism genes, DISCO v1.2 was used on the *Ca*. E. scaldis assembly, with standard parameters [78]. DsrOP homologues were identified in the unfiltered output. DsrJOP homologues were analysed for conserved domain architecture using the online webportal for InterPro [79]. Translated protein sequences of the identified *dsrJOP* genes were aligned with DsrJOP protein sequences, acquired elsewhere [80], using Clustal Omega v1.2.4 with the following parameters: --max-guidetree-iterations=100 --max-hmm-iterations=100 --output-order=tree-order [81]. In addition, the identified DsrP protein sequence was aligned to protein sequences of the NrfD family, acquired elsewhere [82], using Praline with default parameters [82, 83]. Maximum-likelihood phylogenies were calculated using IQtree v1.6.12 with the automatic best-fit model finder and 1000 ultrafast bootstrap replicates [84]. All phylogenies were visualized with FigTree 1.4.4 (http://tree.bio.ed.ac.uk/software/figtree/).

## Results and discussion

### Development of a clonal *Ca*. Electrothrix culture

Genome closure for targeted organisms from an environmental sample is hindered by: (1) the low relative abundance of the target organism, which results in poor coverage; (2) the presence of multiple strains of the target organism, which yields highly similar sequences that hamper the assembly process; and (3) a complex microbial community, which contains species closely related to the target organism, resulting in assembly issues due to interspecies genomic repetition [85–88]. These factors have also likely impeded achieving genome closure of cable bacteria within the genus *Ca*. Electrothrix (see discussion below).

To jointly tackle these three obstacles, we developed a so-called ‘clonal enrichment’ of a *Ca*. Electrothrix strain, which provides a microbial community of strongly reduced complexity that contains a single cable bacterium strain [42, 89]. To create this clonal enrichment, we isolated an individual cable bacterium filament from natural sediment through micromanipulation and transferred this filament to the same sediment, but autoclaved. Autoclaved natural sediment is only partially sterilized, as 16S rRNA V3–V4 gene amplicon analysis revealed that some bacteria remain present, which were mostly spore-forming members of the phylum Bacillota (>96 %; Fig. 2a). We did not completely sterilize the natural sediment medium (e.g. by double autoclaving), as cable bacteria were reported not to grow in such sediment [42].

**Fig. 1.**
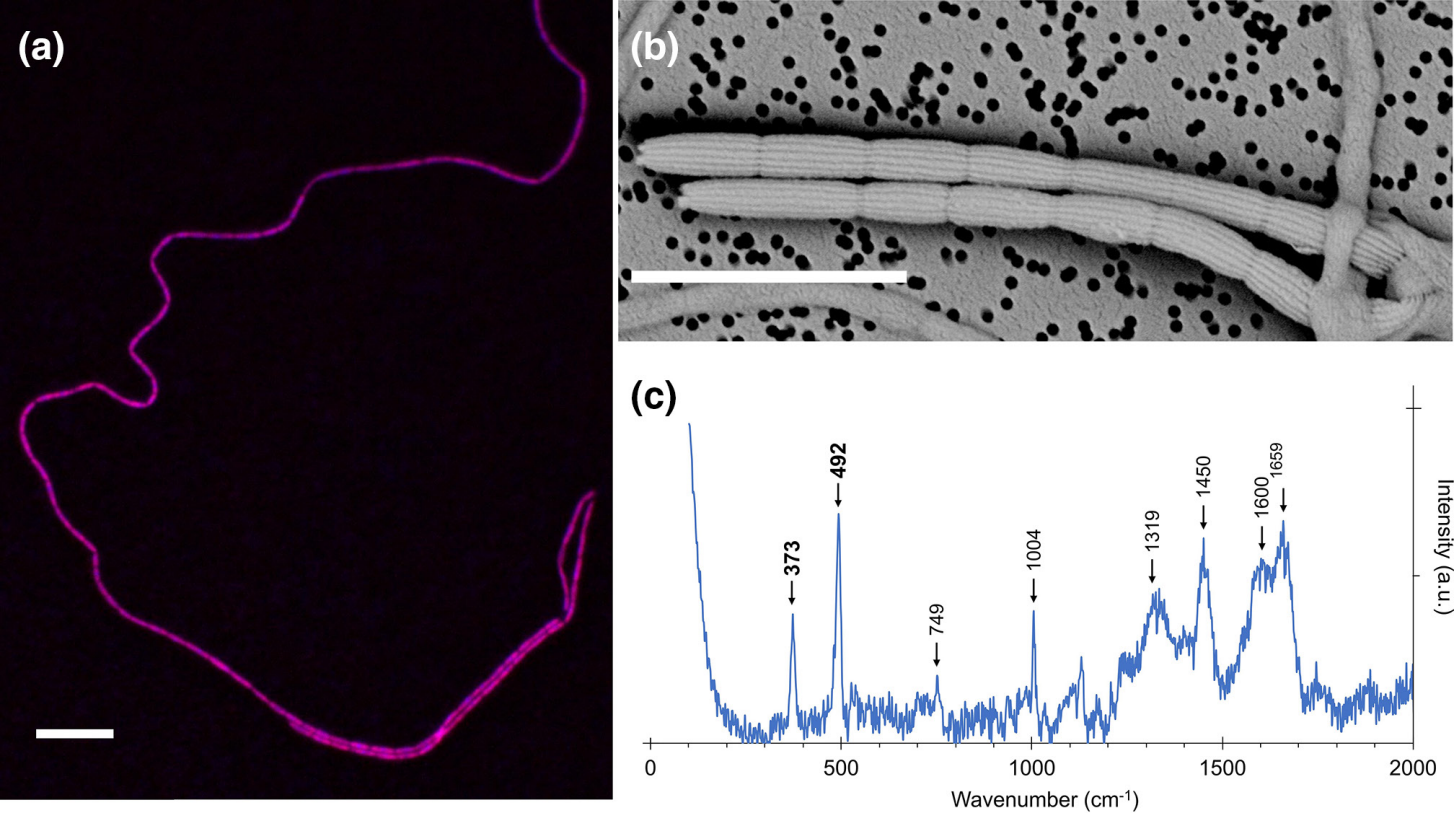
Microscopy and spectroscopy analysis of *Ca*. Electrothrix scaldis filaments from the clonal enrichment culture. (**a**) FISH with probe DSB706, targeting *Desulfobulbaceae*, showing cells in purple, due to overlay of the DSB706 (Cy5, red) and DAPI (blue) images. (**b**) SEM revealed that the filaments displayed 12 ridges that ran in parallel along the longitudinal axis and across cell–cell interfaces. (**c**) Raman spectrum of an intact filament collected with a green laser (532 nm). The low frequency bands at 373 and 492 cm^−1^ (bold) are indicative for the presence of a sulfur–ligated nickel cofactor exclusively found in cable bacteria. Other peaks associated with cytochromes (749, 1319 and 1600 cm^−1^) and protein modes (1004, 1450, 1659 cm^−1^) are also indicated.

In the clonal enrichment culture, filamentous bacteria were observed by light microscopy. These were confirmed to be cable bacteria by FISH (width=0.75±0.09 µm, cell length=3.4±0.5 µm, *n*=10) (Fig. 1a). Additional SEM confirmed that the outer surface of the filaments showed distinct parallel ridges of cable bacteria [24, 90]. Examined filaments (*n*=8) displayed 12 ridges that ran along the entire length of filaments and remained continuous across cell–cell interfaces (Fig. 1b). Raman microscopy further confirmed that the filaments displayed the unique spectroscopic fingerprint that has thus far only been found in cable bacteria (Fig. 1c) [91]. The Raman spectrum shows two distinct peaks at 373 and 492 cm^−1^ that are attributed to vibrational modes from the sulfur-ligated nickel cofactor that sustains long-range conduction in cable bacteria [91]. Finally, 16S V3–V4 rRNA gene amplicon analysis revealed that the enrichment culture contained a single ASV related to cable bacteria (thus rendering it ‘clonal’) with a relative abundance of 17.9 % (thus providing considerable enrichment).

**Fig. 2.**
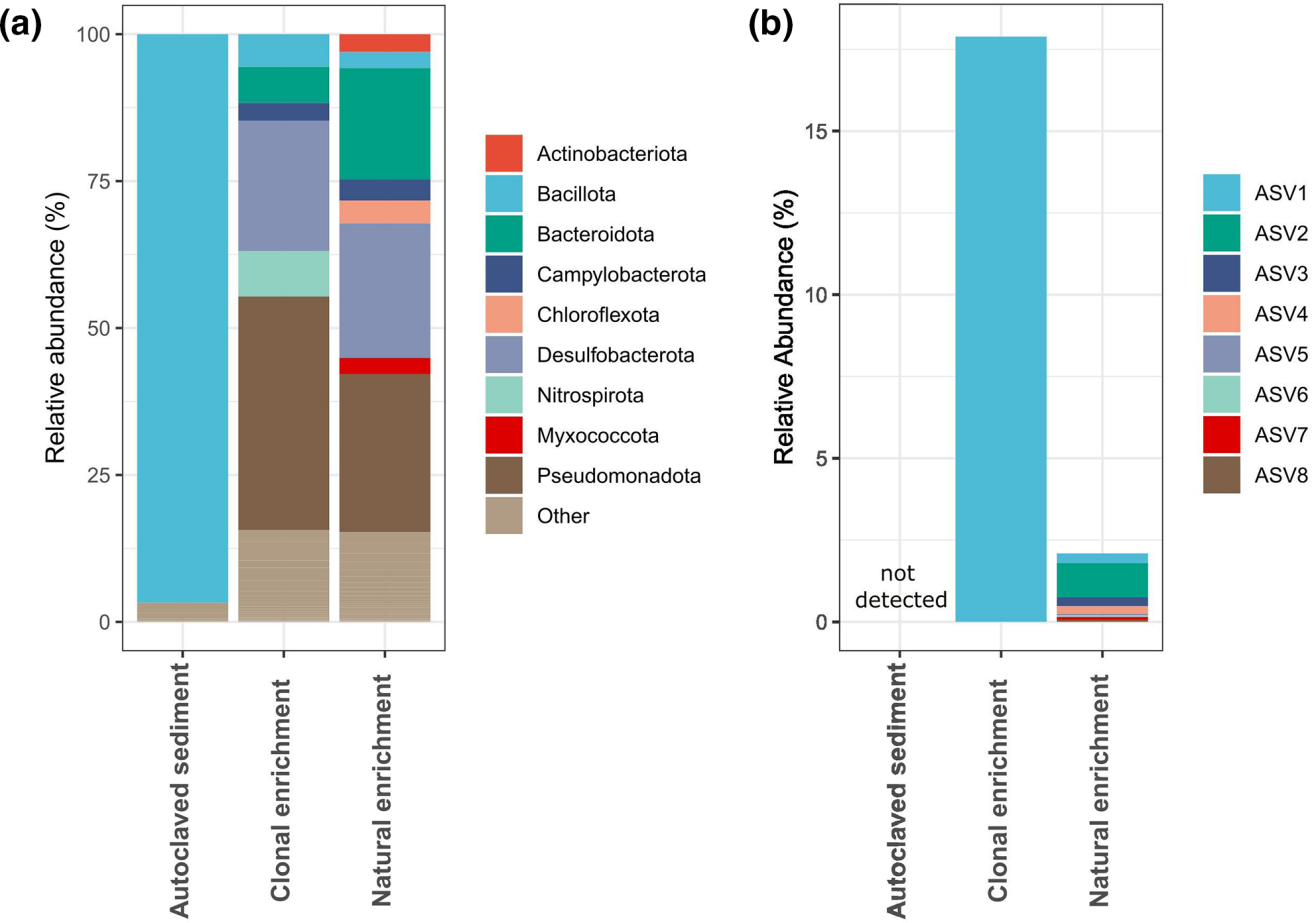
Microbial community composition of autoclaved sediment, the clonal enrichment culture and a natural sediment enrichment culture of cable bacteria. (**a**) Phyla with a relative abundance of >2 %. Cable bacteria belong to the phylum Desulfobacterota. (**b**) Relative abundance of cable bacteria-associated ASVs.

For reference and comparison, we also used a classical, natural enrichment culture from the same sediment for amplicon analysis (i.e. direct incubation of natural sediment, cf [26]). This natural enrichment showed a substantially lower overall relative abundance of cable bacteria compared to the clonal enrichment (2.1 % relative abundance of 16S rRNA gene amplicons). At the same time, it also showed a much higher strain diversity: eight ASVs at >0.01 % abundance were present (Fig. 2b). Moreover, the natural enrichment displayed a far more complex microbial community, as indicated by several diversity indices (e.g. the Shannon diversity index was >6 in the natural enrichment compared to ~4 in the clonal enrichment; Fig. S1, available in the online version of this article). Together, these results demonstrate that our clonal enrichment generated a sediment microbiome with a substantially reduced diversity, and contained a single strain of *Ca*. Electrothrix that was highly enriched in biomass (further referred to as strain GW3-3). This clonal enrichment was used as the starting point for all our sequencing efforts.

### Generation of a closed *Ca*. Electrothrix genome

**Fig. 3.**
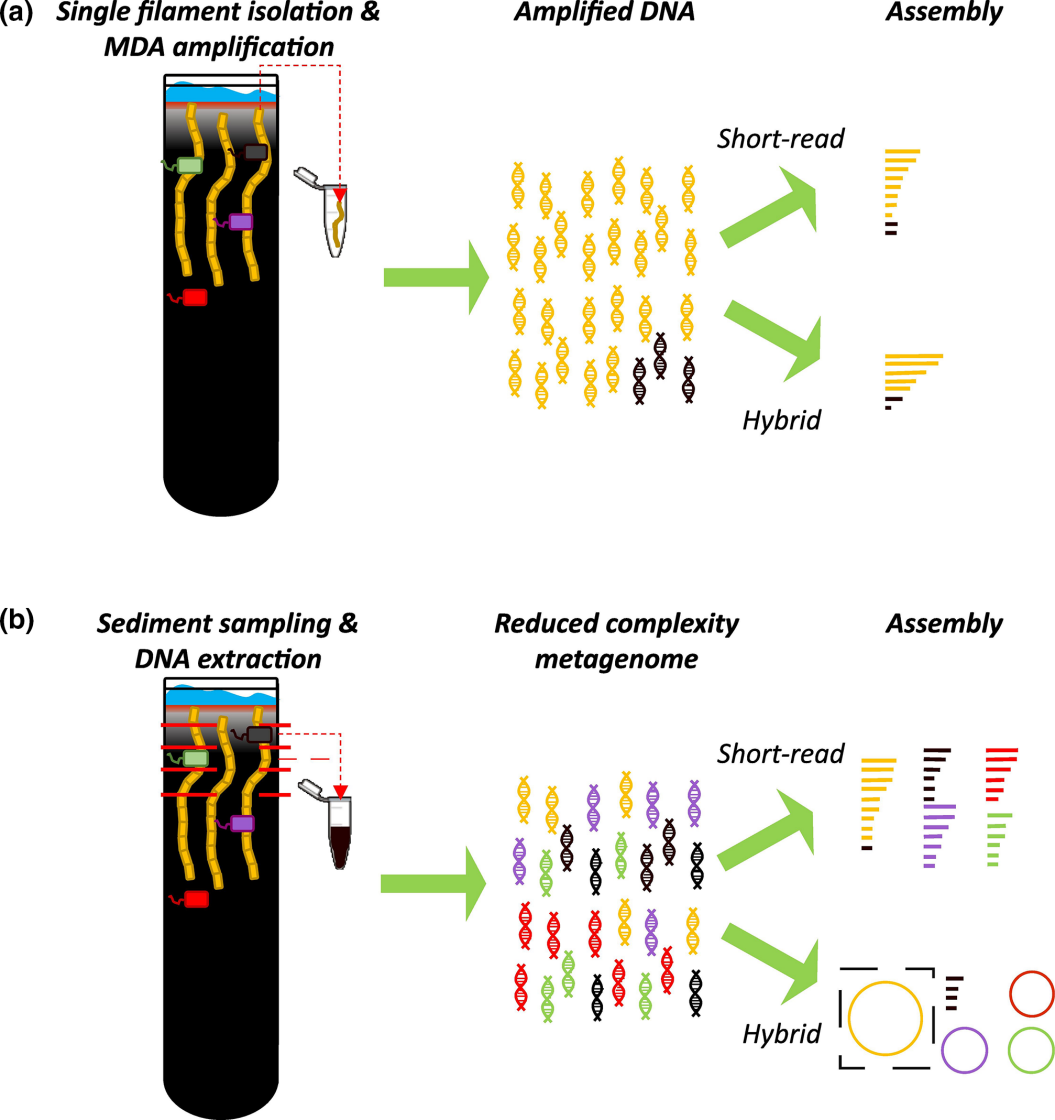
Schematic representation of the approaches for generating genomes from environmental cable bacteria in enrichment cultures. Both approaches were combined with short-read and hybrid (long- and short-read) sequencing and assembly. (**a**) Single-filament amplification approach. Individual cable bacteria filaments were separated from the sediment of a clonal enrichment culture using micromanipulation, following which cells were lysed and the DNA amplified using multiple displacement amplification (MDA). The amplified DNA consists of cable bacteria-derived amplified DNA (yellow) and, potentially, a small fraction of amplified DNA derived from other micro-organisms (black) that remained after repeatedly washing the filament. The resulting assembly was fragmented and binning may be required to remove contamination. (**b**) Metagenome-based approach with a clonal enrichment culture. Extracted genomic DNA contained cable bacteria DNA from a single strain and also non-cable bacteria-derived DNA (other colours) in a reduced complexity metagenome. Assembly using long-read sequencing was the only approach that resulted in closed genomes for the cable bacterium and several other species in the associated microbial community.

Previously, *Ca*. Electrothrix genomes have been generated from a single cable bacterium filament using whole-genome amplification and subsequent short-read sequencing (Fig. 3a) [30, 75]. However, this approach has resulted in assemblies that consist of many contigs (73 to 489 contigs for 6 draft genomes, as reported in [75]. To improve on this, we explored a hybrid genome assembly approach, where we combined short-read, accurate, second-generation sequencing data from Illumina with long-read, less-accurate, third-generation sequencing data from ONT. This hybrid sequencing approach was applied to two DNA retrieval approaches (Fig. 3): single filament genomics and metagenomics (thus enabling a comparison of the performance of these two approaches).

In a first step, we implemented the hybrid sequencing approach to DNA obtained from individual cable bacterium filaments that were retrieved from the clonal enrichments (Fig. 3a). In this ‘single filament amplification approach’, individual filaments were isolated using micromanipulation and lysed, and the DNA was amplified using MDA. Sequencing produced 0.7 Gbp of long-read (Oxford Nanopore) data and 1.2 Gbp of short-read (Illumina 2×150 bp) data (Tables S1, S2). For reference, we compared the hybrid assembly with an assembly that is solely based on short reads (Table 1). Hybrid assembly improved the overall contiguity and assembly size compared to the short read-only assembly. Hybrid assembly resulted in 99 contigs with a genome size of 4.7 Mbp, compared to 317 contigs and a genome size of 4.3 Mbp for the short read-only assembly (Table 1). Additionally, hybrid assembly substantially increased the maximum contig length achieved (473 kb for hybrid vs 101 kb for short read-only assembly; Table 1). Nevertheless, the single-filament amplification approach did not result in a closed genome. We hypothesized that the DNA amplification step was likely the limiting factor for genome closure, as it reduced the potential length of the ONT reads. MDA amplification resulted in fragments with a maximum length of tens of thousands of bp, and required subsequent size selection, which introduced additional DNA shearing. Moreover, it is known that MDA may introduce chimaerism, which could lead to unresolved assemblies or even assembly errors [92].

**Table 1.** Four different approaches were applied for genome assembly of unculturable cable bacteria. Two different approaches for sequencing were combined with two different approaches for assembly. Presented results are for the genome recovered for the target strain GW3-3 (*Ca*. Electrothrix scaldis)

	Single filament amplification		Clonal enrichment metagenome	
**Assembly strategy**	Short read-only	Hybrid	Short-read only	Hybrid
**Average read length (bp**)	317	1723	256	3779
**Assembly size (bp**)	4 044 361	4 914 277	4 830 833	5 089 971
**# of contigs**	384	99	109	1
**Max contig length (bp**)	100 679	472 931	175 611	5 089 971

Hence, we pursued a metagenomic sequencing approach and associated assembly strategy that avoids the need for DNA amplification (Fig. 3b). To this end, we used our clonal enrichment culture and sampled the sub-oxic sediment layer (3–9 mm depth). From this, we extracted sufficient DNA (~5 µg) for hybrid sequencing without the need for amplification. Because the clonal enrichment contained a single cable bacterium-related ASV and the community showed a reduced complexity, we anticipated that this approach would facilitate genome closure.

For this metagenomic sequencing approach, we also implemented a short-read-only assembly as well as a hybrid assembly. Metagenome sequencing produced 9.11 Gbp of long-read (Oxford Nanopore) and 4.3 Gbp of short-read (Illumina MiSeq) data (Tables S1 & S2). The short read-only assembly resulted in 109 contigs (binned, see Methods) and a genome size of 4.8 Mbp for the GW3-3 strain target (Table 1). For the hybrid assembly, we assembled the long reads first in MetaFlye [59] and then polished the resulting nanopore assembly using the short reads with Racon [62]. This allowed us to recover 12 circular MAGs (CMAGs), as well as 11 draft bins with high-quality MAGs (HQ; ≥90 % complete, <5 % contamination) and 22 draft bins with medium-quality MAGs (MQ; ≥50 % complete, <10 % contamination) (Table S3). The CMAGs size ranged from 2.4 to 9.1 Mbp and were confirmed to be circular by manual inspection of mapped reads that overlapped at the contig ends. One of these CMAGs was the cable bacteria target, indicating that the hybrid metagenomic assembly of a clonal enrichment culture resulted in a closed genome (Table 1).

The acquired genome of cable bacterium strain GW3-3 is 5 089 909 bp in size and consists of a single circular contig. It has 4397 protein coding genes and contains three copies of the 16 S-23S-5S rRNA operon. Phylogenomic analysis with 47 cable bacteria genomes from public databases indicates that our closed genome forms a branch in the *Ca*. Electrothrix clade (Fig. S3). ANI comparison and 16S rRNA gene sequence identity with closely related genomes within the genus *Ca*. Electrothrix (Table S4) show that the GW3-3 cable bacterium genome falls outside the species delineation threshold (<98.7 % for 16S rRNA, <95 % for ANI [93, 94]). Thus, strain GW3-3 belongs to a novel species within the genus *Candidatus* Electrothrix. We propose the name *Candidatus* Electrothrix scaldis, after the Scheldt estuary, where the natural sediment for the enrichments was collected.

### Assembly with long reads results in genome closure and native DNA excludes chimaeric artefacts

**Fig. 4.**
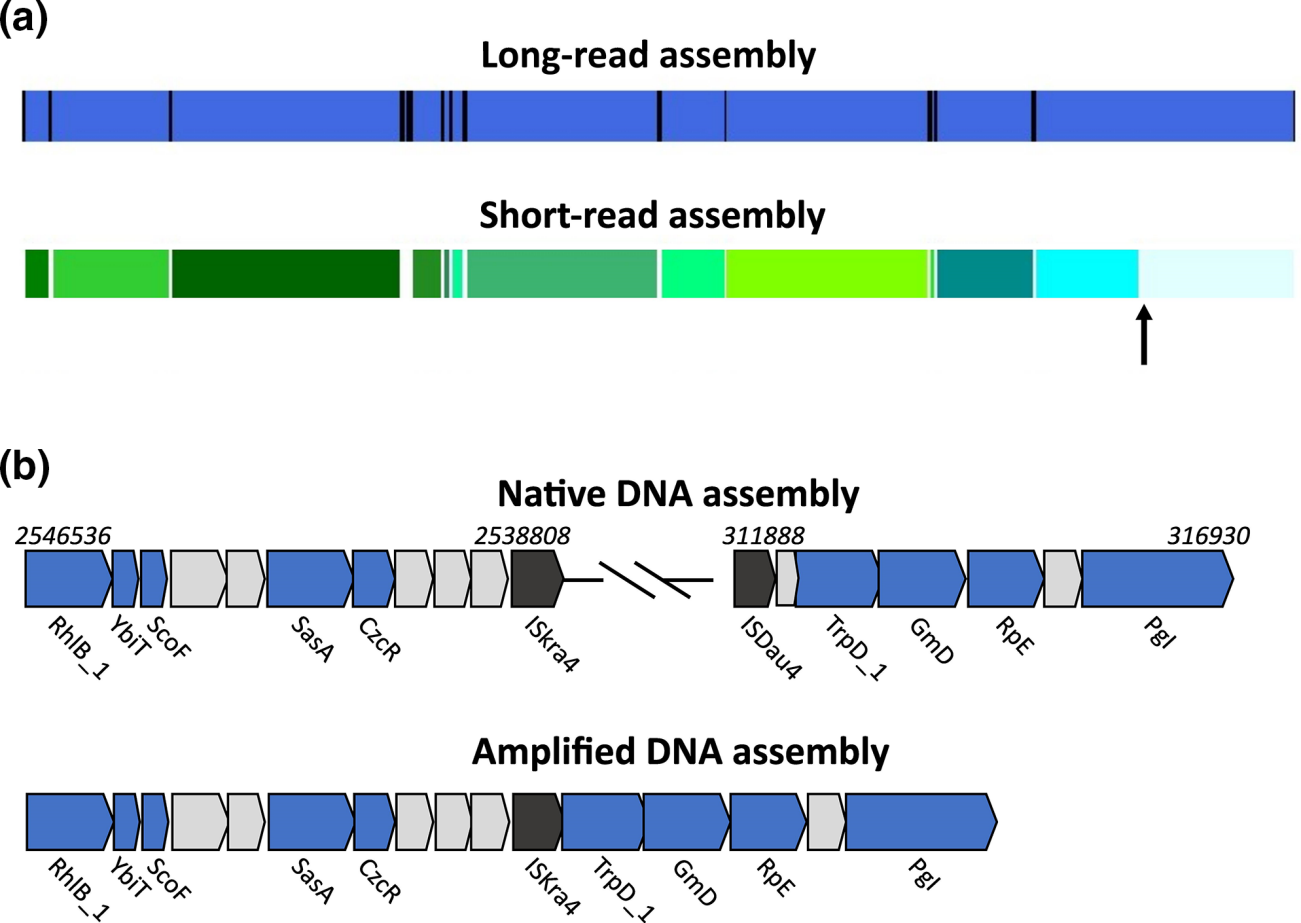
Factors influencing genome closure. (**a**) Contigs generated by short-read assembly (bottom) are aligned to the corresponding part (~500 kbp) of the circular contig generated by long-read assembly (top). In both cases, DNA was acquired without amplification (native). Contigs of the SR assembly (green) seem to ‘break’ almost exclusively near intragenomic repeats found in the closed genome (black bars in the LR assembly). On rare occasions, the SR assembly breaks when no intragenomic repeat is present (black arrow), which could be explained by potentially unidentified intergenomic repetition in the metagenome. (**b**) Example of an artificial translocation that is observed in the assembly based on single filament-amplified DNA (bottom), when compared to a similar region in the closed genome acquired from native DNA (top). Both assemblies are made with long reads. The uninterrupted locus found with amplified DNA is spatially separated in distant parts of the closed genome (locations are indicated in italics). Thus, two parts corresponding to different regions of the amplified DNA LR assembly are artificially stitched together, near the position of a non-repeating IS of the ISKra4 family (black). Hypothetical proteins are indicated in grey and genes with predicted functions are indicated in blue.

Long-read sequencing was essential to obtain the closed genome of *Ca*. E. scaldis, as an assembly made from just the metagenomic Illumina MiSeq data resulted in a fragmented genome assembly with 109 contigs (Table 1). Insertion sequences (ISs) form a common repetitive element within microbial genomes [95], and this is also the case for *Ca*. E. scaldis. There are 120 predicted putative ISs in the genome (3.4 % of the total genetic code). To further investigate whether these ISs are repetitive, we identified all ‘repeats’ (two sequences >250 bp in length that are at least >99 % identical) in *Ca*. E. scaldis (Table S5). Indeed, ISs contribute to the vast majority of repeats in the genome (86 out of 97 repeats in total; Table S5), which range from 545 to 5215 bp in size (median 1625 bp; Table S5). These repeats are the main cause of assembly fragmentation, as almost all contigs of the short read-assembled genome break near these intragenomic repetitive elements (Fig. 4a). This observation illustrates the value of long reads to span repetitive sequences during genome assembly [1].

Our results also suggest that the amplification step in the single-filament amplification approach resulted in chimaeric artefacts. When contigs from the hybrid single filament assembly (generated from amplified DNA) were aligned to the final closed genome (generated from native DNA), artificial translocations were identified (Fig. 4b). These are likely chimaeric artefacts introduced by the linkage of non-contiguous homologous regions during amplification [92]. Thus, we conclude that two aspects of our metagenomic assembly were essential to achieve genome closure: (1) the use of long reads to bridge repeats (of which the majority are ISs) and (2) the use of native DNA to omit the risk of introducing chimaeric artefacts by DNA amplification.

### Metagenomic genome closure is linked to high abundance and low strain heterogeneity

**Fig. 5.**
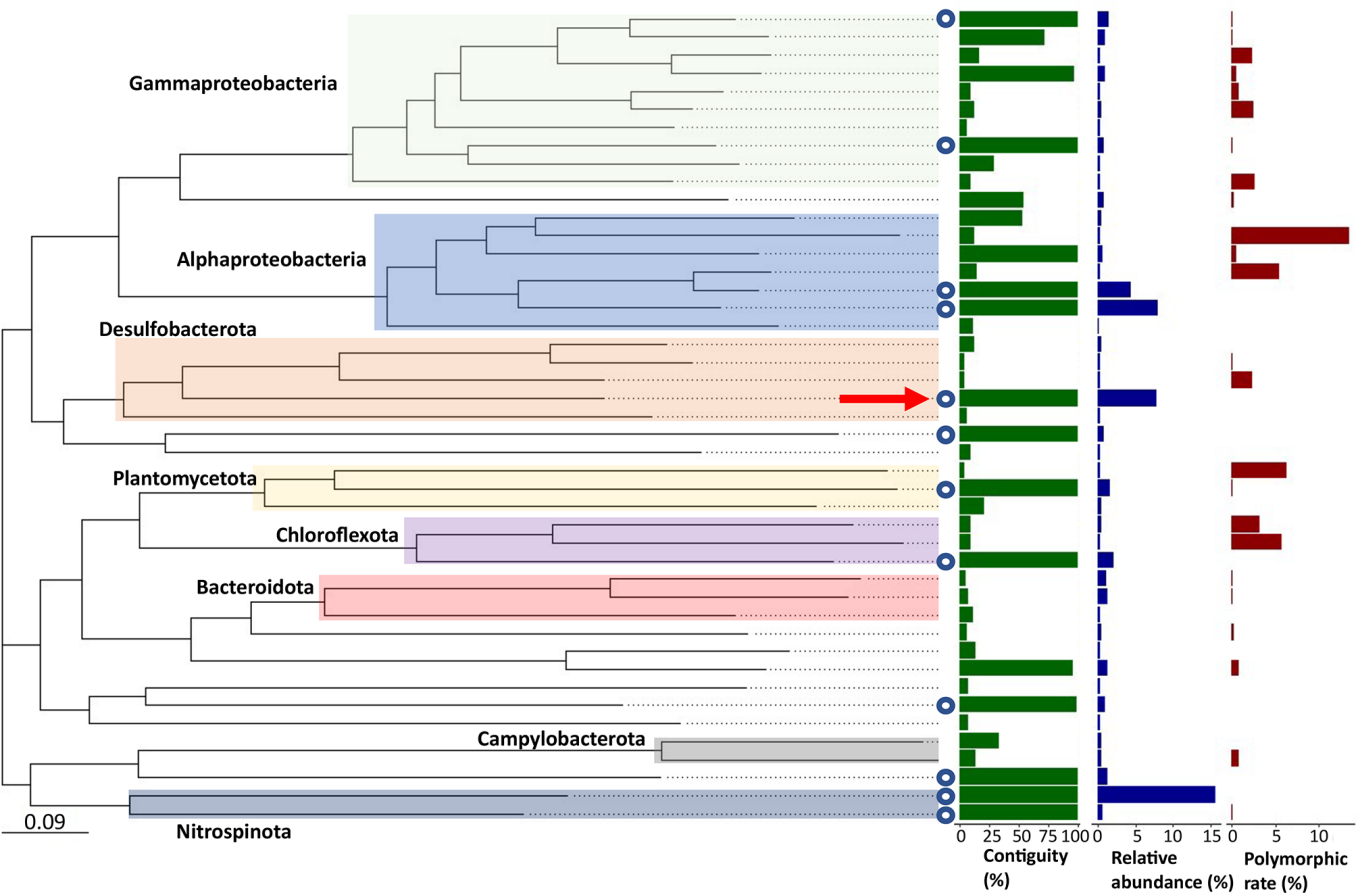
Phylogenomic tree of the clonal enrichment assembled community. Closed genomes are indicated with circles. Selected phyla and classes are indicated. The closed cable bacterium genome (red arrow) is affiliated to the Desulfobacterota. Closed genomes generally represent microbial community members with a higher relative abundance (blue bars) and lower strain heterogeneity (red bars). Moreover, higher contiguity (green bars) is correlated with lower strain heterogeneity in all metagenome assembled genomes.

From the clonal enrichment, we recovered 62.4 % of the sequenced community in 42 MAGs. Together, the 12 CMAGs accounted for 46 % of all sequence reads, with individual CMAGs having a relative abundance ranging from 0.6–15.7 % (Table S3), indicating that most CMAGs have a relatively high abundance. However, high abundance is not the only factor affecting genome circularity. There were several other bins (HQ-MAGs) that did not circularize, even though these bins had a higher relative abundance (0.8 %–1.3 %) than some of the CMAGs in the assembled metagenome (Table S3). Strain-level heterogeneity has previously been reported to severely hamper the assembly of genomes generated from short-read sequencing [87, 96, 97]. To verify whether this is also the case here, we determined the strain heterogeneity of the CMAGs and HQ-MAGs by calculating the average polymorphic site rate, a measure for strain heterogeneity in metagenomic samples [69]. The polymorphic site rate of CMAGs was 0–0.14 %, which is considerably lower than the 0.11–2.49 % for the HQ-MAGs (Table S3). Moreover, in all acquired MAGs, high assembly contiguity appeared to correlate with high relative abundance and low strain heterogeneity (Fig. 5). Thus, our data confirm the strong impact of strain heterogeneity on assembly contiguity. Here, through clonal enrichment of cable bacteria, we were able to reduce the strain heterogeneity to only one strain (0 % polymorphic rate), while retaining a high relative abundance (7.9 % of sequenced reads). This emphasizes the value of clonal enrichment culturing for genome closure.

### New insights into carbon, oxygen, nitrogen and sulfur metabolism of cable bacteria

With 5.09 Mbp, the genome of *Ca.* E. scaldis is larger than the 37 incomplete genomes of *Ca*. Electrothrix previously described, and also larger than available *Ca*. Electronema genomes [30, 41, 75] (Fig. S4). Of the 4397 protein coding genes, 1109 genes (24%) are not found in any of the available genomes of *Ca*. Electrothrix. However, not all of these ‘single genes’ are specific to *Ca*. E. scaldis, as some genes were likely not identified in other available *Ca*. Electrothrix genomes due to a lack of genome closure. Indeed, if we functionally assign these 1109 genes to COG categories [77], we find genes with COG functional categories that are expected to be present in all *Ca*. Electrothrix members, as they relate to ‘household’ genes (Fig. S5). It seems unlikely that these genes are strain specific. This hence stresses the importance of genome closure for comparative genomics and core genome analysis.

The closed genome of *Ca*. E. scaldis enables new insights into the electrogenic metabolism of cable bacteria, thus complementing previous genomic studies [41, 75]. In terms of carbon metabolism, currently available *Ca*. Electrothrix genomes encode the complete Wood–Ljundahl pathway, pentose phosphate pathway and tricarboxylic acid cycle [41, 75]. Remarkably, the metalloprotein enolase, which catalyses the penultimate step in glycolysis and Entner–Doudoroff pathways, was reported missing in the available (incomplete) genomes of *Ca*. Electrothrix, as well as in the closed genomes of *Ca*. Electronema aureum and *Ca*. Electronema halotolerans [41, 75]. However, in the closed genome of *Ca*. E. scaldis obtained here, enolase is putatively encoded (Table S6; 78% amino acid sequence identity with enolase of *Desulfatiglans anilini*). In terms of synteny, the enolase gene is distantly located from the other glycolysis genes in the genome, but it is not positioned near regions that are hard to assemble, such as repeats. Thus, we decided to retroactively search for enolase in all available *Ca*. Electrothrix genomes and found one other genome encoding enolase: *Ca*. Electrothrix SY2 [41]. It is noteworthy that *Ca*. Electrothrix SY2 has the highest completeness (98.8%) of all non-closed *Ca*. Electrothrix genomes (Fig. S4), and that *Ca*. Electrothrix SY2 is distantly related to *Ca*. E. scaldis (Fig. S3). Therefore, we hypothesize that enolase and the complete glycolysis pathways could be present more frequently in the genus *Ca*. Electrothrix than we can currently detect with the available genomic data. Additional closed genomes are paramount to further clarify this issue.

Cable bacteria use oxygen as their main terminal electron acceptor [24], and high rates of oxygen consumption in aquatic sediments have been attributed to the activity of cable bacteria [98]. Furthermore, expressed on a cellular basis, cable bacteria appear to display among the highest oxygen consumption rates recorded within prokaryotes [99, 100]. Paradoxically, initial genome analysis of cable bacteria showed little capacity for oxygen reduction, as a terminal cytochrome c oxidase was lacking [75]. Moreover, the observed biosynthetic activity of oxidizing cells was found to be very limited, which would imply that oxygen reduction is not coupled to energy conservation [75, 99]. However, in the genomes of *Ca*. Electrothrix communis and *Ca*. Electrothrix laxa, genes coding for all four subunits of the aa_3_-type cytochrome c oxidase are present [41, 75]. Here, we find that *Ca*. E. scaldis possesses genes coding for similar subunits (Table S6). For the putative CoxA subunit, the copper- and haem-binding residues are well conserved, as well as key residues involved in the D and K proton relay channels [101–103] (Fig. S6). This suggests that *Ca*. E. scaldis could possess a suitable system for oxygen reduction, although dedicated physiological rate measurements are needed to experimentally verify this. Additional closed genomes are needed to verify how widespread the aa_3_-type cytochrome c oxidase is in other members of the genus Electrothrix.

Experimental evidence indicates that cable bacteria can perform dissimilatory nitrate reduction to ammonium [104, 105]. In the closed genome of *Ca*. Electronema aureum, the periplasmic NapAB protein complex has been suggested to perform nitrate reduction to nitrite [41, 104]. Canonical nitrite reductases from the nir or nrf type were not detected, yet a multiheme cytochrome (pOOC) adjacent to the nap operon was suggested as a potential enzyme that could catalyse the reduction of nitrite to ammonium [104]. Here, we find that a similar full-length nap operon is present in the genome of *Ca*. E. scaldis, including *napB*, which was previously reported to be undetected in incomplete *Ca*. Electrothrix genomes [41]. Similarly, the gene putatively encoding the multiheme cytochrome for nitrite reduction (pOOC) was detected in very close proximity to the nap gene cluster (Table S6). This indicates that members of the genus *Ca*. Electrothrix have the ability to perform dissimilatory nitrate reduction to ammonium.

The closed genome of *Ca*. E. scaldis also provides new insights into the sulfur metabolism of cable bacteria. Cable bacteria oxidize sulfide within the deeper layers of the sediment, and this so-called anodic sulfide oxidation has been proposed to be performed by reversal of the canonical sulfate reduction pathway [24, 75]. Hitherto, only a handful of putative genes from the Dsr pathway have been identified in previous studies, encoding the DsrAB, DsrC, DsrN and DsrMK proteins [41, 75]. This could imply that cable bacteria have either a minimal set of Dsr genes, as typically found in ancient sulfate reducers [80], or alternatively, that some Dsr genes have escaped detection (e.g. because of genome incompleteness). More complex Dsr pathways are found in other sulfur-reducing and sulfur-oxidizing bacteria, where genes coding for the DsrJOP subunits were recruited to form the DsrMKJOP membrane complex [80]. This complex acts as an oxidoreductase catalysing the reduction of DsrC-trisulfide with electrons from the quinone pool generating a protein gradient over the membrane [106]. The exact role of the DrsJOP unit is unclear, since it is not essential in some organisms, but it has been proposed that DsrMKJOP attains a higher energetic efficiency than DsrMK, by improved quinone cycling or proton pumping [106, 107]. In cable bacteria genomes, a gene coding for a potential DsrJ homologue was previously identified, but *dsrOP* genes were not detected [75]. The closed *Ca*. E. scaldis genome confirms the presence of the putative *dsrJ* gene, but also indicates the presence of genes putatively encoding DsrOP (Table S6). The gene putatively coding for DsrJ, a periplasmic c-type cytochrome, lies in synteny with *dsrMK*. The gene putatively encoding DsrO is predicted to have the characteristic TAT signalling peptide as well as domains to bind 4Fe–4S clusters [108]. The putatively encoded homologue of DsrP is predicted to be a membrane protein resembling NrfD with 10 transmembrane helices [108]. Dsr proteins can be phylogenetically categorized as either reductive-type proteins or oxidative-type proteins [80]. To assess which type the putative DsrJOP homologues belong to, we performed a phylogenetic analysis using the expansive dataset of DsrJOP homologues described by Neukirchen *et al*. [80]. Interestingly, our phylogenetic analysis indicates that the putative DsrJOP homologues found in *Ca*. E. scaldis cluster with reductive-type proteins (Figs S7, S8, S9). As the putative DsrP homologue in *Ca*. Electrothrix. scaldis formed a long branch, we speculated that the putative DsrP homologue could be phylogenetically more closely related to other members of the NrfD protein family [82]. To assess this, we performed a phylogenetic analysis with the DsrP homologue found in *Ca*. E. scaldis and NrfD family protein sequences provided by Duarte *et al*. [82], which demonstrated that the NrfD domain found in the putative DsrP homologue is clearly related to the canonical DsrP (Fig. S10). Furthermore, unlike in the closely related *Desulfogranum* species, the *dsrOP* genes are not located in the same operon as the d*srMKJ* genes (Table S6), which could explain why they were previously undetected in incomplete *Ca*. Electrothrix genomes [75]. Indeed, the putative *dsrOP* genes detected in *Ca*. E. scaldis seem to have been acquired horizontally, as DsrOP homologues of the *Desulfogranum* species form a distantly related branch (Figs S8 and S9). In addition to the genes encoding for the DsrMKJOP complex, two other *dsr* genes are found in the closed *Ca*. E. scaldis genome that were not previously reported in cable bacteria: *dsrD*, an allosteric activator of DsrAB [109, 110] and *dsrT*, which, based on sequence homology, might regulate gene expression of the DsrMKJOP complex [111]. All in all, our data indicate that the genetic potential for sulfur metabolism in cable bacteria encodes a more complex pathway than previously thought.

## Conclusion

With this study, we provide a methodology to generate closed genomes of unculturable cable bacteria, which relies on the development of a clonal enrichment culture and metagenomic hybrid sequencing. We systematically investigated the factors that promote genome closure. The achievement of a clonal culture provides reduced strain-level heterogeneity and increases the relative abundance of the target organism, which both facilitate genome closure. Moreover, we show that sequencing of native DNA excludes artefacts introduced by amplification, while long reads are essential to bridge the many repeats present in the genome. This methodology resulted in the first closed genome within the genus *Ca*. Electrothrix, for a novel species with the proposed name *Ca*. Electrothrix scaldis. The availability of a closed genome provides increased insight into the electrogenic metabolism of cable bacteria, revealing genes involved in glycolysis, oxygen reduction, nitrate reduction and sulfur metabolism that were previously missed in incomplete assemblies.

### Taxonomic proposal

Description of ‘*Candidatus* Electrothrix scaldis’ sp. nov.: ‘*Candidatus* Electrothrix scaldis’ (scal′dis, from L. n. Scaldis, Scheldt estuary; N.L. adj. g. scaldis). This taxon is represented by strain GW3-3. The complete protologue can be found in Table S7.

## Supplementary Data

Supplementary material 1

Supplementary material 2
