# Supplementary material for: Closing the genome of unculturable cable bacteria using a combined metagenomic assembly of long and short sequencing reads
Source: Microb Genom. 2024 Feb 20;10(2):001197. doi: 10.1099/mgen.0.001197 (PMC10926707; doi:10.1099/mgen.0.001197)
Supplement: Supplementary material 1 [file mgen-10-1197-s001.pdf]

Supplementary Material

Closing the genome of unculturable cable bacteria using a combined metagenomic assembly of long and short sequencing reads

Anwar Hiralal[1], Jeanine S. Geelhoed[1], Silvia Hidalgo-Martinez[1], Bent Smets[1], Jesper R. van Dijk[1], and Filip J.R. Meysman[1,2]

1. Geobiology research group, University of Antwerp, Antwerp, Belgium.
2. Department of Biotechnology, Delft University of Technology, Delft, The Netherlands.

corresponding author: filip.meysman@uantwerpen.be; F.J.R.Meysman@tudelft.nl

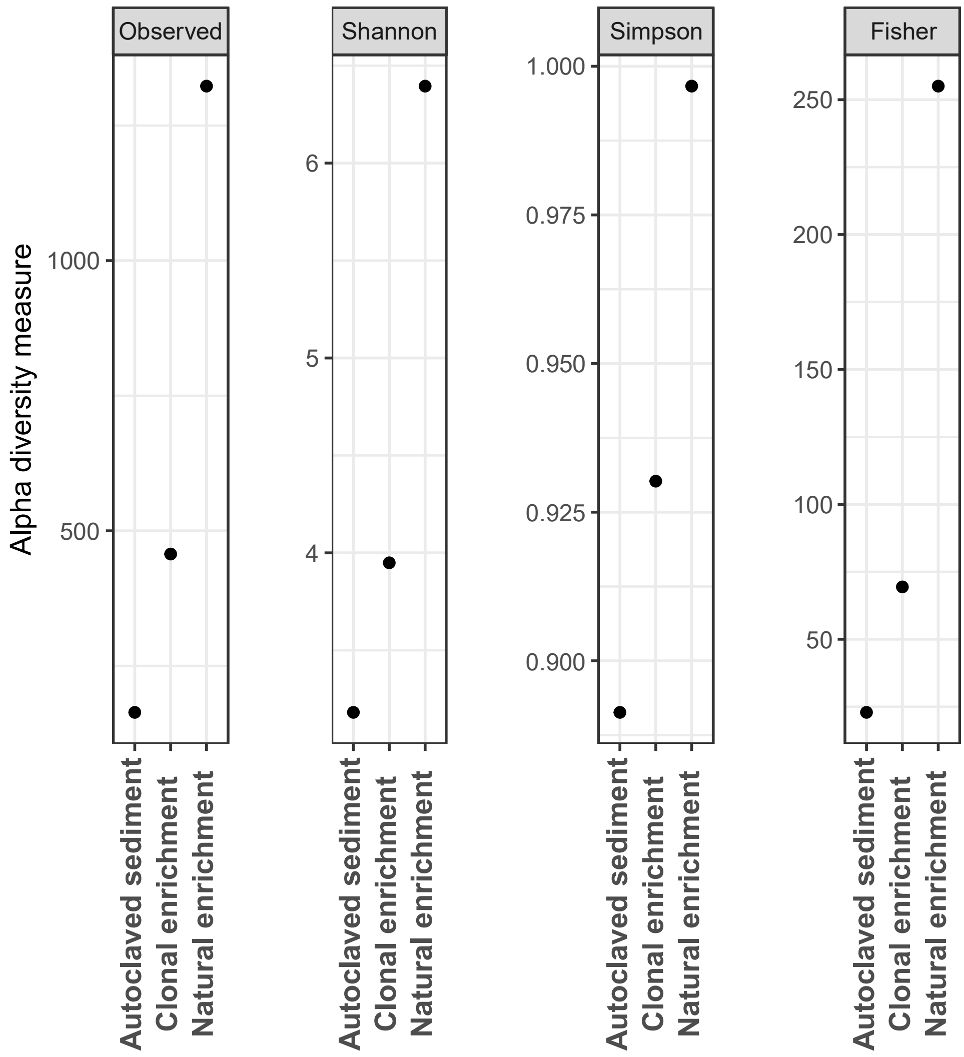

Figure S1: Alpha diversity measures of observed, Shannon, Simpson and Fisher indices (autoclaved sediment 29542 reads, clonal enrichment 50233 reads, natural enrichment 45407 reads).

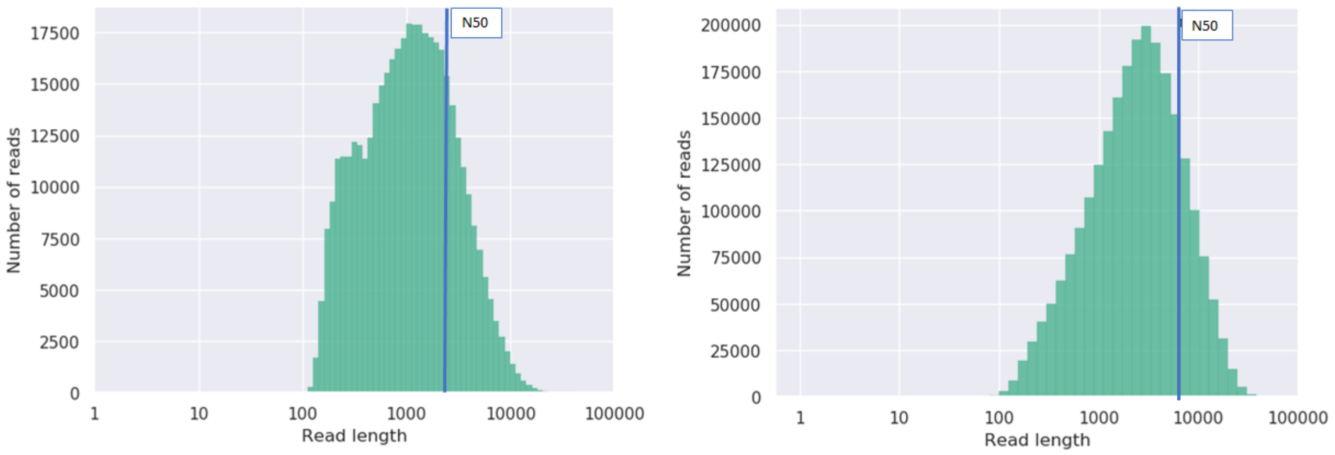

Figure S2: Read length distribution of ONT sequencing reads. **A**: Log-scaled read length histogram of the single filament, MDA amplified, DNA sample (N50=2939). **B**: Log-scaled read length histogram of the clonal enrichment metagenome (N50=6376). Figures obtained using NanoPlot (De Coster et al., 2018).

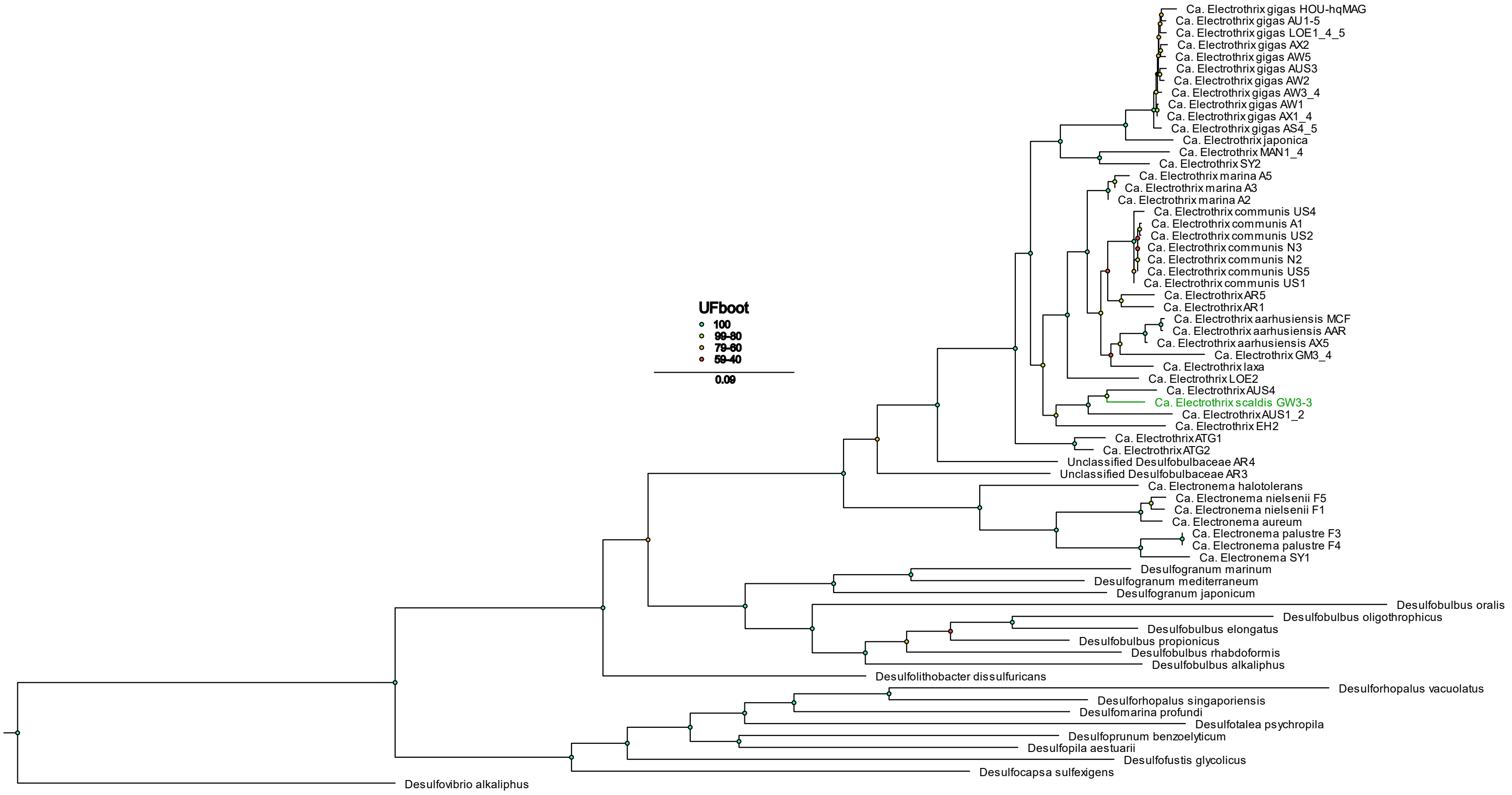

Figure S3: Phylogenomic tree of cable bacteria genomes and selected reference genomes. Cable bacteria genomes were acquired from several studies (Geelhoed et al., 2023; Kjeldsen et al., 2019; Sereika et al., 2023). assemblies of cultured species of the Desulfobulbia class were downloaded from the NCBI database and were used as reference genomes. The circular genome generated in this study is highlighted in green. Ultrafast bootstrap (UFBoot) values are indicated above the scalebar. The tree was generated using GTDB-Tk v1.6.0 and IQtree v1.6.12 with best-fit model LG+F+R4 (Chaumeil et al., 2020; Nguyen et al., 2015). Accession numbers for all genomes can be found in Table S8.

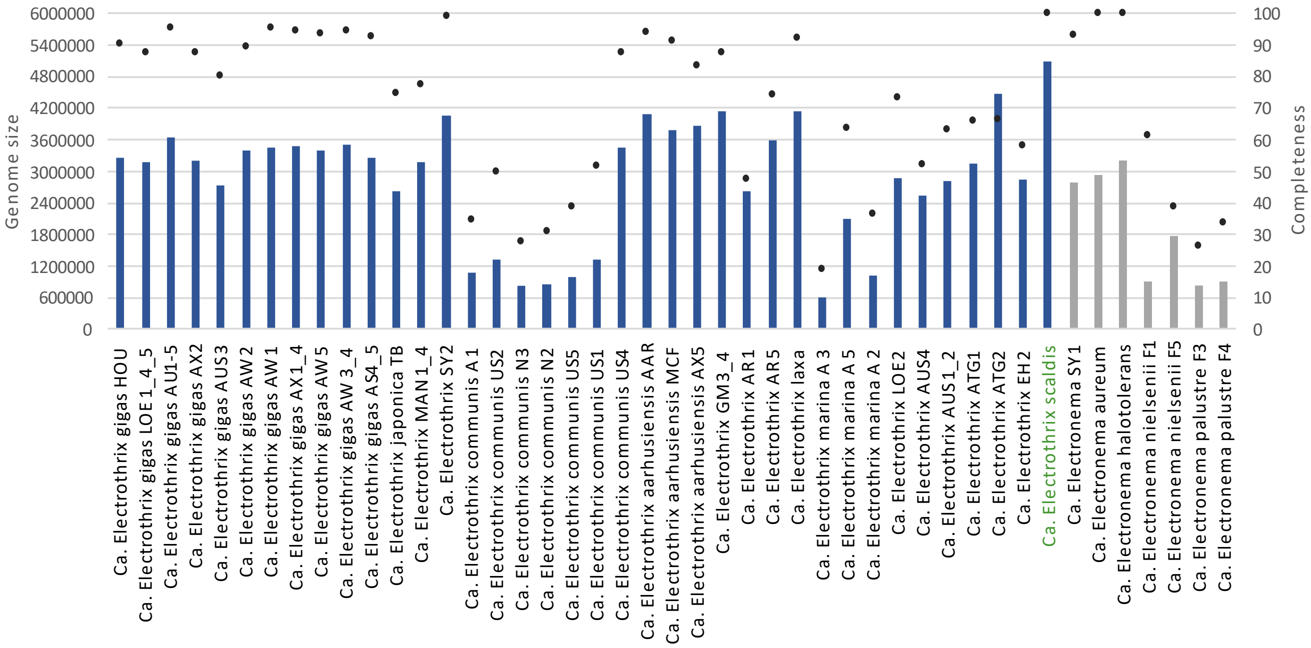

Figure S4: Genome completeness and genome size of all available *Ca*. Electrothrix and *Ca*. Electronema genomes. Genome sizes (0.6Mbp-5.09Mbp) are indicated in bars and color coded for the two different genera. Genome completeness (26.8-100%) is indicated with black dots. The *Ca*. Electrothrix scaldis genome is indicated with green. Closed genomes are set at 100%.

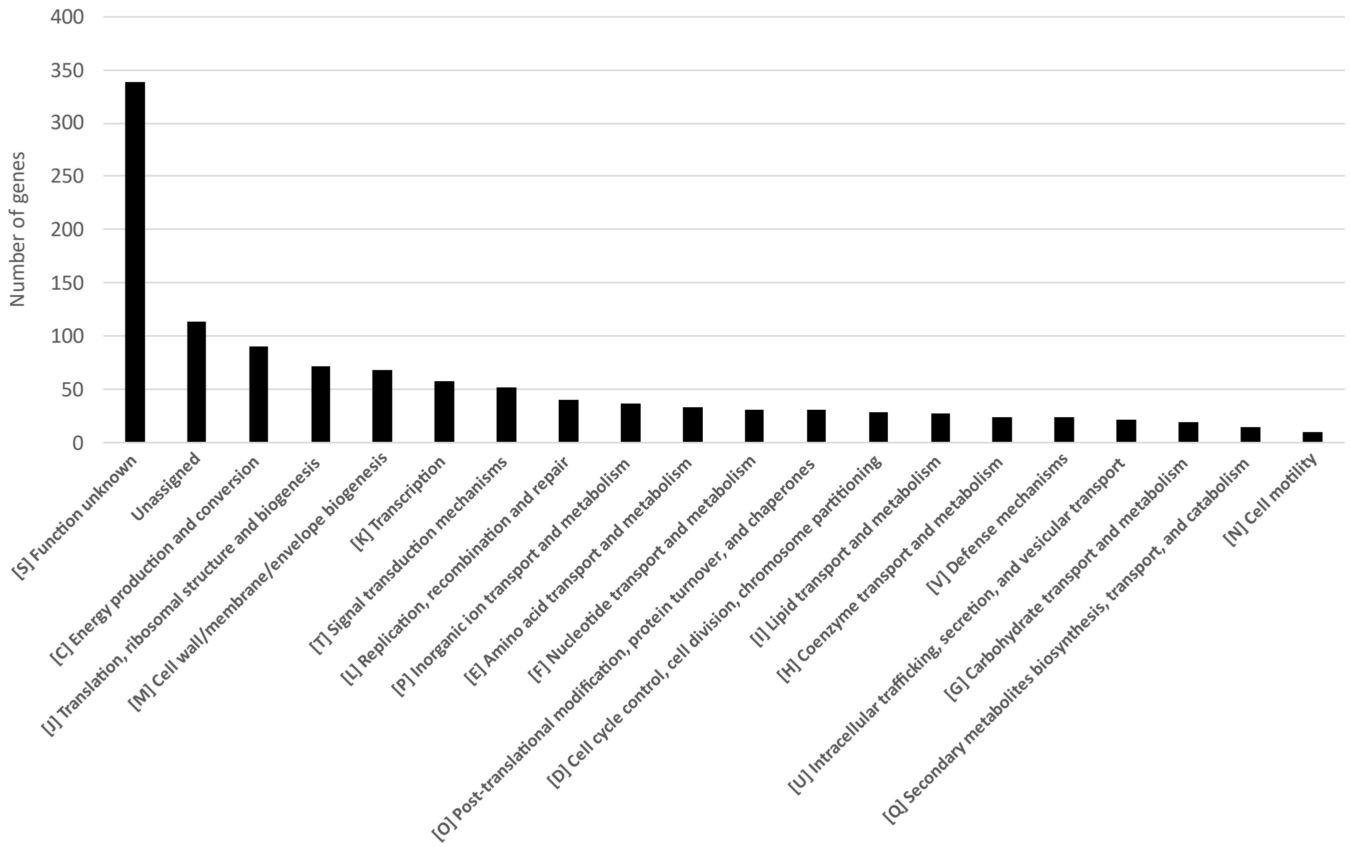

Figure S5: COG functionality assignment for the 1109 genes uniquely identified in *Ca*. Electrothrix scaldis. Assigned using EggNOG-mapper (Cantalapiedra et al., 2021).

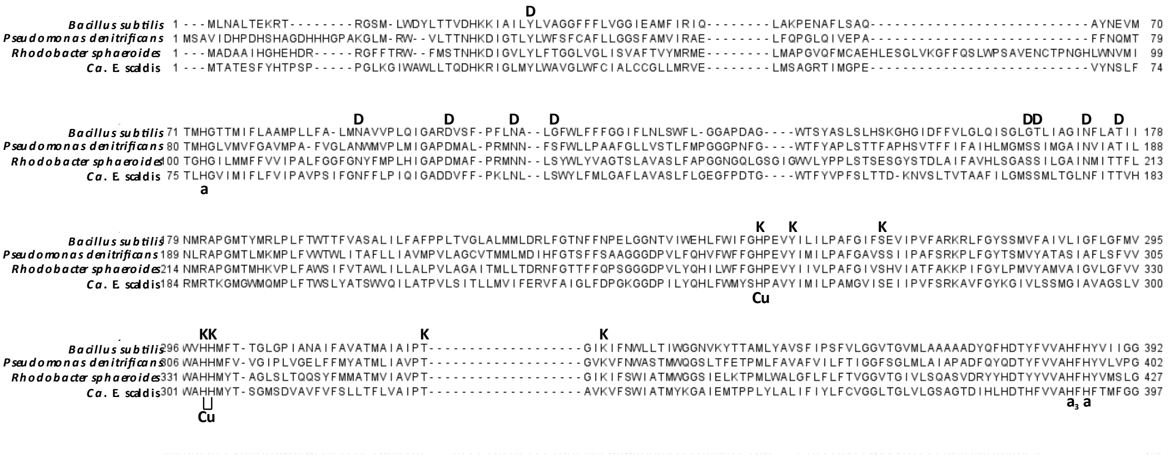

Figure S6: MSA highlighting conserved residues in the aa_3-type cytochrome c oxidase subunit 1. Cu= copper binding site, a3 = haeme a3, a= haeme a, D= residues implicated in D-channel, K= residues implicated in K-channel.

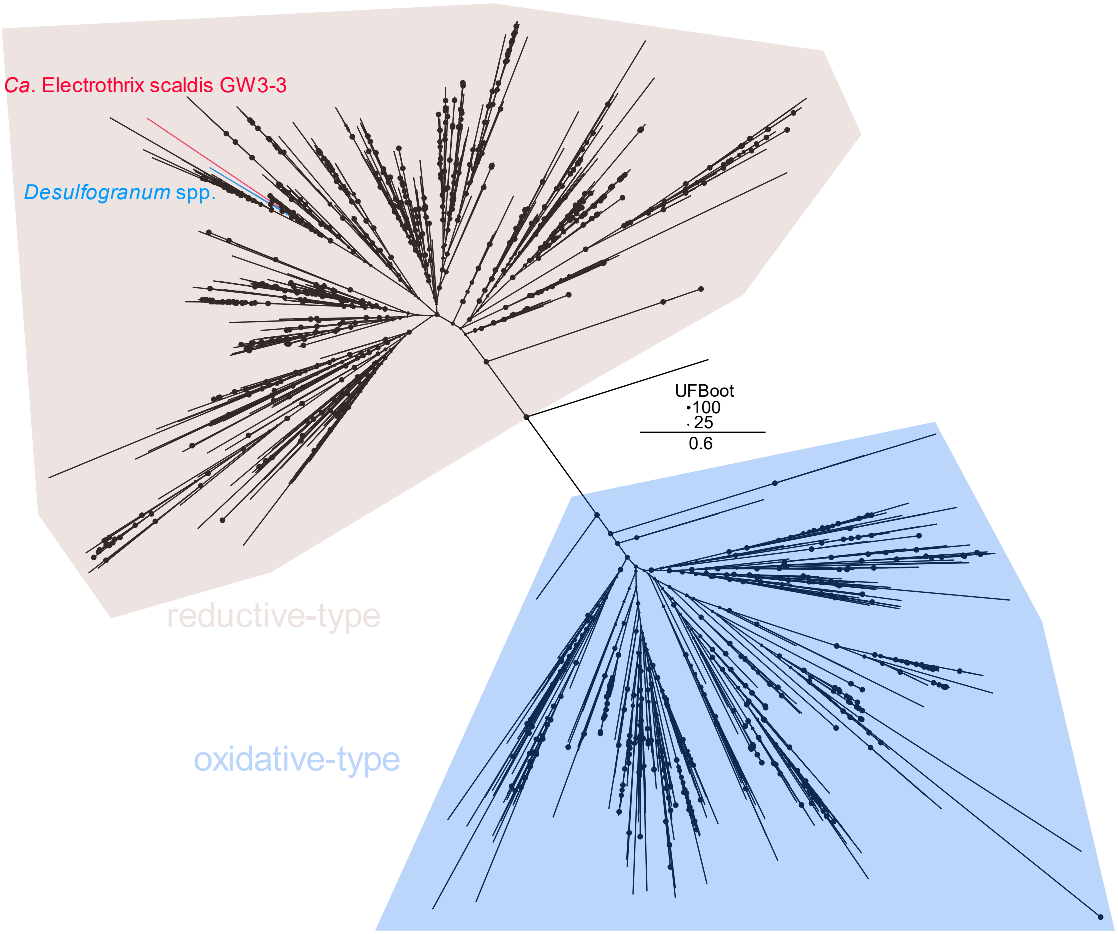

Figure S7: Maximum likelihood phylogeny of DsrJ proteins (model LG+I+G4). Cable bacteria DsrJ homologs are indicated in red branches. Black circles indicate ultrafast bootstrap values. DsrJ protein sequences were acquired from Neukirchen et al., 2023.

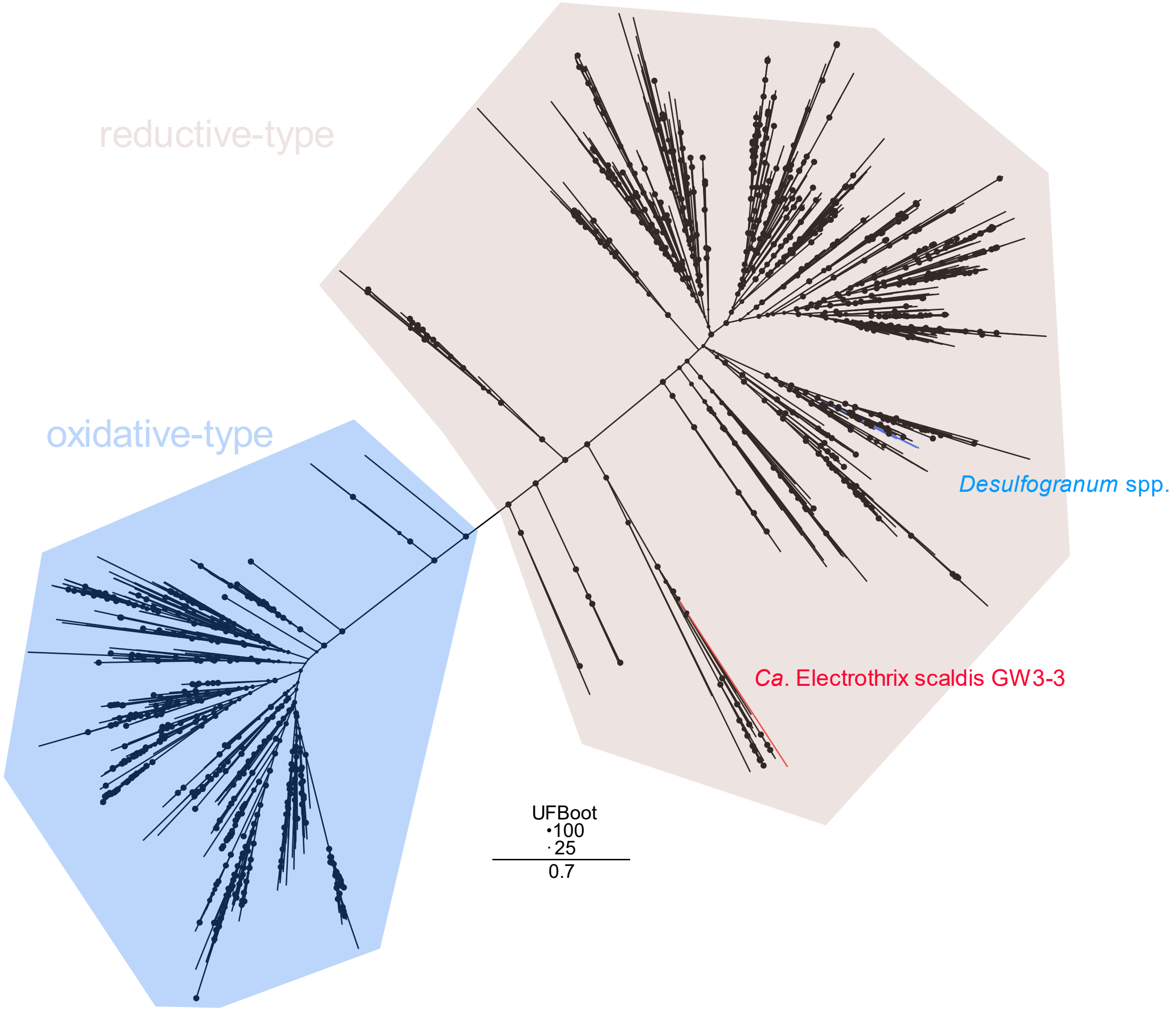

Figure S8: Maximum likelihood phylogeny of DsrO proteins (model LG+I+G4). Cable bacteria DsrO homologs are indicated in red branches. Black circles indicate ultrafast bootstrap values. DsrO protein sequences were acquired from Neukirchen et al., 2023.

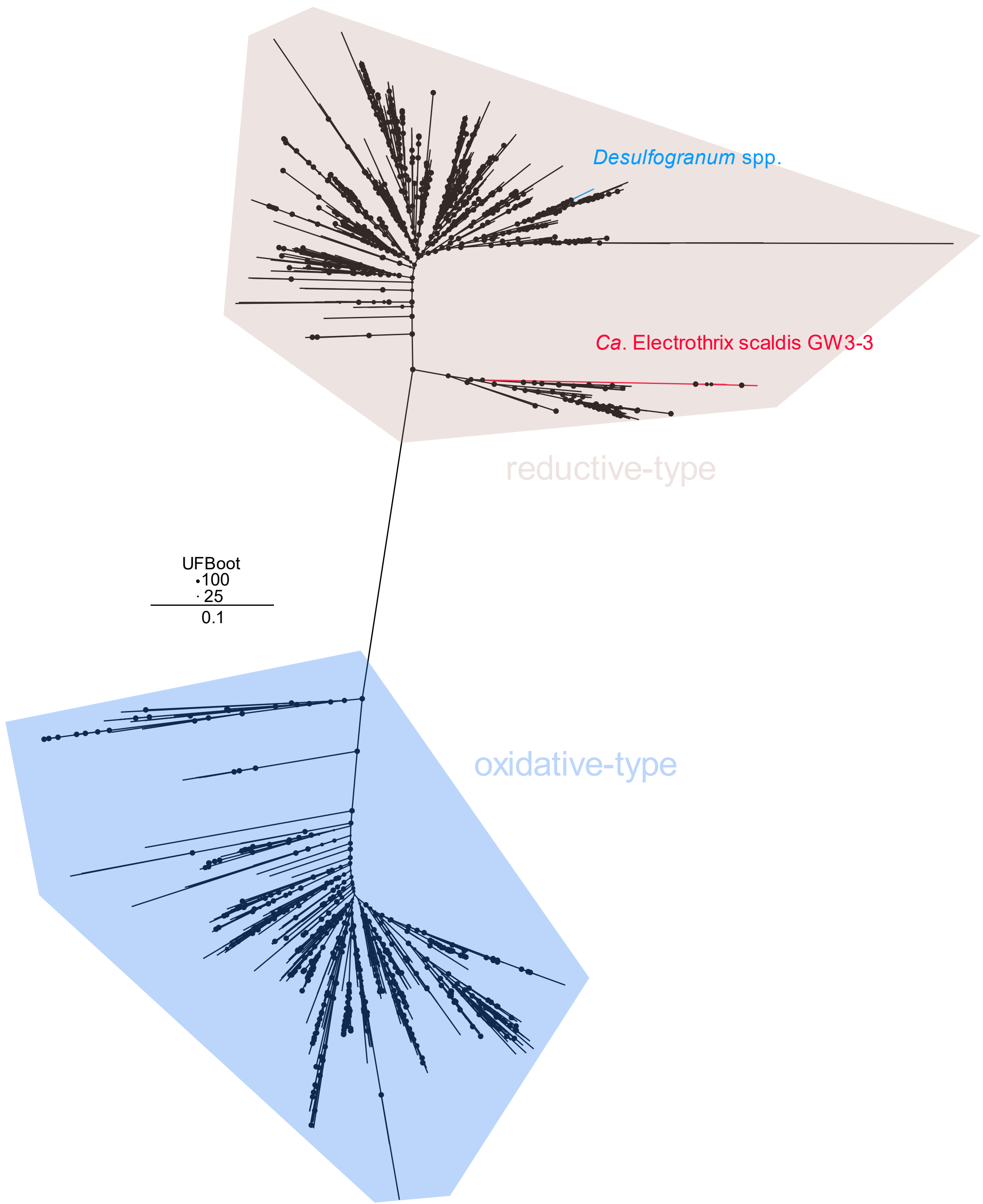

Figure S9: Maximum likelihood phylogeny of DsrP proteins (model LG+I+G4). Cable bacteria DsrP homologs are indicated in red branches. Black circles indicate ultrafast bootstrap values. DsrP protein sequences were acquired from Neukirchen et al., 2023.

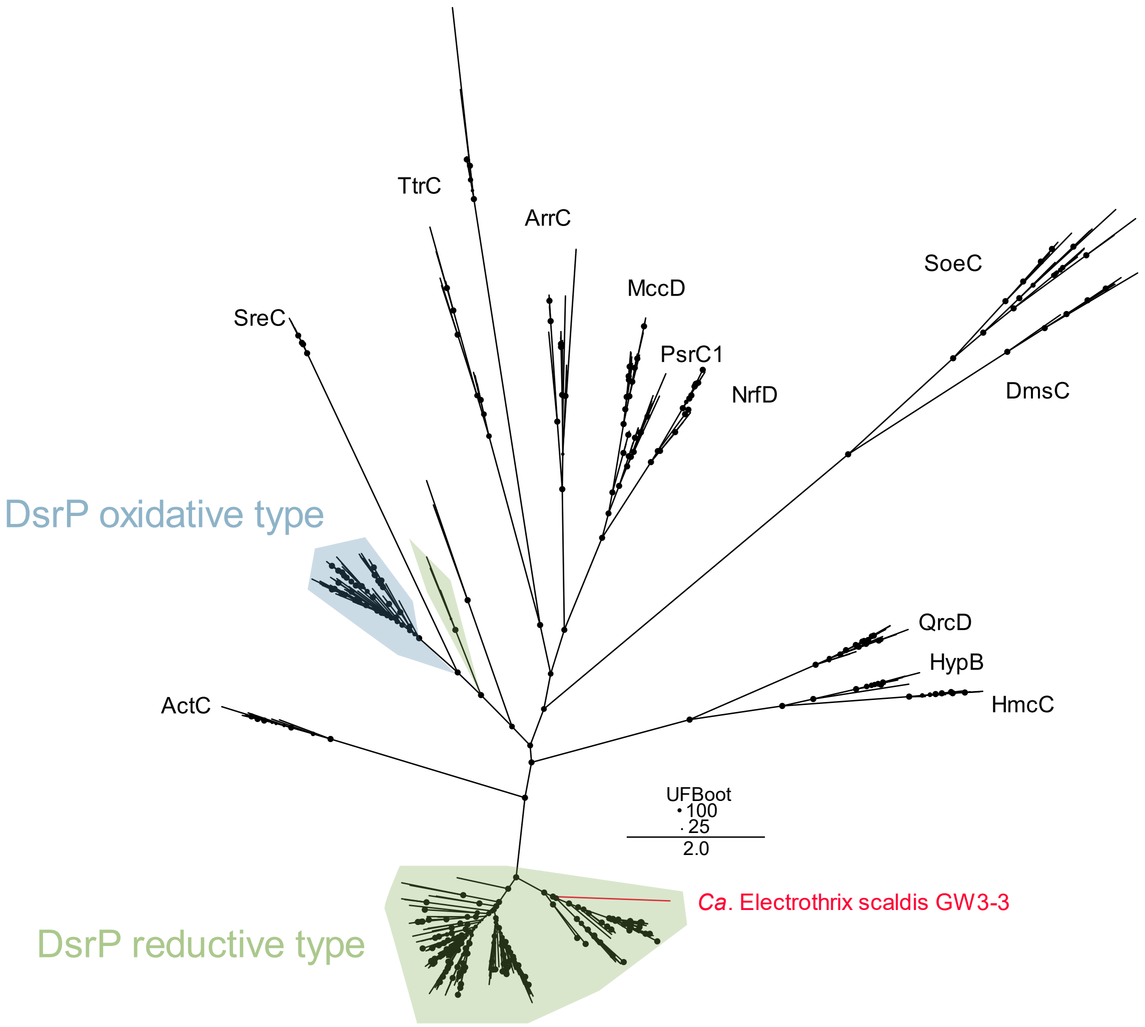

Figure S10: Maximum likelihood phylogeny of NrfD family proteins (model LG+I+G4). Cable bacteria DsrP homologs are indicated in red branches. Black circles indicate ultrafast bootstrap values. NrfD family protein sequences were acquired from Duarte et al., 2018.

Table S1: Oxford Nanopore MinION sequencing overview for the single filament amplified cable bacteria genome and the clonal enrichment metagenome.

	Single filament amplification	Clonal enrichment metagenome
**Nanopore flow cell**	FLO-MIN106	FLO-MIN106
**Sequencing kit**	SQK-RBK004	SQK-LSK109
**Barcoding kit**	SQK-RBK004	EXPNBD114
**Total bases after qc (bp)**	727,108,233	9,106,362,329
**Total reads after qc**	421,794	2,409,489
**Average read length for sample (bp)**	1,723	3,779
**N50 (bp)**	2939	6376

Table S2: Illumina HiSeq and MiSeq sequencing overview for the single filament amplified cable bacteria genome and the clonal enrichment metagenome.

	Single filament amplification	Clonal enrichment metagenome
**Sequencing platform**	Illumina HiSeq	Illumina MiSeq
**Total paired reads after qc**	41,854,263	5,320,548
**Total paired bases after qc (bp)**	14,915,930,605	3,724,000,935
**Average insert size (bp)**	317	257

Table S3: Bin quality, taxonomic identification, relative abundance, polymorphic rate and contiguity.

Table S3 is provided as a separate excel file

Table S4: 16S rRNA gene sequence identity and average genome nucleotide identity (ANI) between *Candidatus* Electrothrix scaldis and the most closely related cable bacteria.

**Species**	***Ca.* Electrothrix scaldis GW3-3**	
	**16S rRNA**	**ANI**
***Ca.* Electrothrix AUS1_2**	93.38%	81.39%
***Ca.* Electrothrix AUS4**	96.59%	85.34%
***Ca.* Electrothrix EH2**	93.96%	80.59%
***Ca.* Electrothrix LOE2**	94.93%	79.74%
***Ca.* Electrothrix marina A2**	96.91%	80.67%

Table S5: repeats in the closed genome of *Ca.* Electrothrix scaldis GW3-3.

Rep. nr.	Repeat sequence	Start	End	Length
1	IS1380	13593	15266	1673
2	IS1634	40138	41648	1510
3	IS1380	121471	123085	1614
4	IS66	122934	123940	1006
5	IS1380	163681	165320	1639
6	IS3	268894	269515	621
7	IS701	295210	296762	1552
8	ISKRA4	311865	313406	1541
9	ISL3	350181	351591	1410
10	ISKRA4	356182	357870	1688
11	ISKRA4	359750	361217	1467
12	IS1380	361752	363363	1611
13	IS4	391661	392938	1277
14	IS1380	537094	538769	1675
15	hypothetical gene cluster 3	563246	565093	1847
16	IS1380	600878	602504	1626
17	ISKRA4	607409	608979	1570
18	IS5	608980	610116	1136
19	IS5	625793	627455	1662
20	IS1380	632386	634061	1675
21	IS1380	727497	729172	1675
22	ISL3	779513	780925	1412
23	IS1380	1016688	1018365	1677
24	ISKRA4	1231960	1233653	1693
25	IS3	1261696	1262451	755
26	IS3	1264287	1264832	545
27	ISKRA4	1305733	1307304	1571
28	IS5	1307868	1309516	1648
29	ISKRA4	1309937	1311629	1692
30	IS1634	1311556	1312279	723
31	IS1380	1319898	1321574	1676
32	16S-23S-5S rRNA	1378429	1383642	5213
33	IS1380	1434232	1435907	1675
34	ISKRA4	1745744	1747314	1570
35	ISAZO13	1844836	1846128	1292
36	ISAZO13	1847494	1848957	1463
37	IS3	1898865	1900151	1286
38	IS1380	1946232	1947857	1625
39	IS1380	1949795	1951468	1673
40	IS1380	1951670	1953285	1615
41	hypothetical gene cluster 3	1976404	1978274	1870
42	ISKRA4	1983080	1984772	1692
43	IS1380	1986121	1987796	1675
44	IS1380	2232508	2234183	1675
45	IS1380	2254509	2256122	1613
46	IS1380	2261064	2262739	1675
47	IS1380	2294230	2295842	1612
48	IS66	2352311	2353781	1470
49	IS1380	2353781	2355455	1674
50	IS1380	2355955	2357568	1613
51	ISKRA4	2367507	2369078	1571
52	IS1380	2369079	2370645	1566
53	IS630	2423509	2424714	1205
54	IS3	2451335	2452621	1286
55	IS1380	2487536	2489149	1613
56	IS1380	2492626	2494273	1647
57	ISL3	2504744	2506154	1410
58	IS5	2509156	2510803	1647
59	ISL3	2602435	2603845	1410
60	ISL3	2647362	2648772	1410
61	IS3	2692320	2693594	1274
62	IS1380	2737792	2739310	1518
63	IS5	2940098	2941761	1663
64	IS1380	3079835	3081511	1676
65	IS5	3104150	3105813	1663
66	IS1380	3119572	3121250	1678
67	IS1380	3147500	3149177	1677
68	ISKRA4	3331205	3332902	1697
69	IS1380	3584891	3586566	1675
70	IS1380	3596813	3598488	1675
71	IS1380	3611257	3612932	1675
72	ISKRA4	3808772	3810342	1570
73	hypothetical gene cluster1	3810806	3812470	1664
74	IS66	3812637	3814107	1470
75	hypothetical gene cluster2	3841655	3842426	771
76	hypothetical gene cluster1	3933923	3935557	1634
77	ISKRA4	3951827	3953398	1571
78	IS1380	4015593	4017268	1675
79	hypothetical gene cluster1	4126957	4128529	1572
80	ISKRA4	4126958	4128528	1570
81	IS1380	4165482	4167104	1622
82	IS1380	4336393	4338070	1677
83	16S-23S-5S rRNA	4358273	4363487	5214
84	16S-23S-5S rRNA	4364416	4369631	5215
85	IS1380	4636738	4638413	1675
86	IS5	4638413	4640075	1662
87	IS1380	4645494	4647169	1675
88	IS1380	4846196	4847871	1675
89	ISKRA4	4847884	4849576	1692
90	IS1380	4858530	4860206	1676
91	ISL3	4907030	4908442	1412
92	ISKRA4	4999951	5001521	1570
93	hypothetical gene cluster 1	5002369	5004002	1633
94	hypothetical gene cluster2	5004003	5004834	831
95	IS1380	5018052	5019665	1613
96	IS701	5019687	5020802	1115
97	IS1380	5025090	5026765	1675

Table S6: Locus tag of genes of selected metabolic genes found in *Candidatus* Electrothrix scaldis GW3-3.

Gene	Locus Tag	Protein Number	Annotation	Pathway
dsrM	SD837_21840	4368	Sulfite reduction-associated complex DsrMKJOP protein DsrM	sulfur metabolism
dsrK	SD837_21835	4367	Sulfite reduction-associated complex DsrMKJOP protein DsrK	sulfur metabolism
dsrJ	SD837_21830	4366	Sulfite reduction-associated complex DsrMKJOP protein DsrJ	sulfur metabolism
dsrO	SD837_06975	1395	Sulfite reduction-associated complex DsrMKJOP protein DsrO	sulfur metabolism
dsrP	SD837_06980	1396	Sulfite reduction-associated complex DsrMKJOP protein DsrP	sulfur metabolism
dsrA	SD837_02355	471	Dissimilatory sulfite reductase alpha subunit	sulfur metabolism
dsrB	SD837_02350	470	Dissimilatory sulfite reductase beta subunit	sulfur metabolism
dsrC	SD837_03860	772	Dissimilatory sulfite reductase related protein	sulfur metabolism
dsrD	SD837_02345	469	Dissimilatory sulfite reductase system component, protein DsrD	sulfur metabolism
dsrT	SD837_21845	4369	Dissimilatory sulfite reductase system component, protein DsrT	sulfur metabolism
dsrN	SD837_22365	4473	Cobyrinate a,c-diamide synthase	sulfur metabolism
glk	SD837_15065	3013	glucokinase	glycolysis
pgi	SD837_01450	290	glucose-6-phosphate isomerase	glycolysis
pfk	SD837_06950	1390	6-phosphofructokinase 1	glycolysis
fbp	SD837_18075	3615	fructose-1,6-bisphosphatase	glycolysis
aldo	SD837_02515	503	fructose-bisphosphate aldolase	glycolysis
tpi	SD837_05580	1116	triose-phosphate isomerase	glycolysis
gapdh	SD837_03440	688	glyceraldehyde-3-phosphate dehydrogenase	glycolysis
pgk	SD837_05585	1117	phosphoglycerate kinase	glycolysis
pgm	SD837_06095	1219	phosphoglycerate mutase	glycolysis
eno	SD837_09660	1932	enolase	glycolysis
ppdk	SD837_06925	1385	pyruvate phosphate dikinase	glycolysis
napA	SD837_18100	3620	Periplasmic nitrate reductase (NapA)	nitrogen metabolism
napB	SD837_18085	3617	Periplasmic nitrate reductase, electron transfer subunit (NapB)	nitrogen metabolism
napD	SD837_18105	3621	Periplasmic Nitrate reductase, chaperone subunit (NapD)	nitrogen metabolism
napF	SD837_18110	3622	Periplasmic Nitrate reductase, Ferredoxin-type protein (NapF)	nitrogen metabolism
napG	SD837_18095	3619	Periplasmic Nitrate reductase, Ferredoxin-type protein (NapG)	nitrogen metabolism
napH	SD837_18090	3618	Periplasmic Nitrate reductase, Ferredoxin-type protein (NapH)	nitrogen metabolism
pOOC	SD837_18120	3624	periplasmic multiheme cytochrome	nitrogen metabolism
coxA, ccoA	SD837_04400	880	cytochrome c oxidase subunit 1	oxygen reduction
coxB, ccoB	SD837_04415	883	cytochrome c oxidase subunit II	oxygen reduction
coxC, ccoC	SD837_04405	881	cytochrome c oxidase subunit III	oxygen reduction
coxD, ccoD	SD837_04410	882	cytochrome c oxidase subunit IV	oxygen reduction

Table S7: Protologue table for *Candidatus* Electrothrix scaldis.

**Species name**	*Candidatus* Electrothrix scaldis
**Genus name**	*Candidatus* Electrothrix
**Specific epithet**	scaldis
**Type strain**	GW3-3
**Type species of the genus**	*Candidatus* Electrothrix aarhusiensis
**Genus status**	Candidatus
**Species etymology**	Description of '*Candidatus* Electrothrix scaldis' sp. nov.: "*Candidatus* Electrothrix scaldis" (scal´dis, from L. n. Scaldis, Scheldt river; N.L. adj. g. scaldis).
**Species status**	sp. nov.
**Assembly project**	PRJNA1030987
**Genome accession number**	GCA_033584155.1
**Genome topology**	Circular
**Genome Size (bp)**	5089909
**GC%**	48.17%
**Country of origin**	The Netherlands
**Region of origin**	Zeeland
**Sample source**	Salt marsh sediment
**Geographical location**	Rattekaai (Eastern Scheldt river)
**Latitude**	51.439051°N
**Longitude**	4.168504°E
**Sample Depth**	-15-0 cm
**Assembly method**	Hybrid
**Sequencing technology**	Oxford Nanopore R9.4.1 and Illumina MiSeq
**Binning**	Not performed
**Assembly software used**	Flye 1.2
**Habitat**	Intertidal zone (coastal habitat)
**Miscellaneous, extraordinary features relevant for the description**	Circular genome obtained from a metagenomic sample of a clonal cable bacterium enrichment

Table S8: Accession numbers of cable bacteria genomes and selected reference genomes.

**Species**	**Genome accession**
Ca. Electronema aureum AUR-cMAG	GCA_942492785.1
Ca. Electronema halotolerans	GCA_942493095.1
Ca. Electronema nielsenii F1	GCA_026122975.1
Ca. Electronema nielsenii F5	GCA_026122915.1
Ca. Electronema palustre F3	GCA_026122955.1
Ca. Electronema palustre F4	GCA_026122935.1
Ca. Electronema SY1	GCA_011391865.1
Ca. Electrothrix A5	GCA_004028495.1
Ca. Electrothrix aarhusiensis AAR	GCA_942491045.1
Ca. Electrothrix aarhusiensis AX5	GCA_022766085.1
Ca. Electrothrix aarhusiensis MCF	GCA_004028505.1
Ca. Electrothrix AR1	GCA_022765785.1
Ca. Electrothrix AR5	GCA_022765745.1
Ca. Electrothrix AR–5	GCA_022765745.1
Ca. Electrothrix ATG1	GCA_022765905.1
Ca. Electrothrix ATG2	GCA_022765965.1
Ca. Electrothrix AUS1_2	GCA_022765865.1
Ca. Electrothrix AUS4	GCA_022765925.1
Ca. Electrothrix communis A1	GCA_004028485.1
Ca. Electrothrix communis N2	GCA_026122895.1
Ca. Electrothrix communis N3	GCA_026122875.1
Ca. Electrothrix communis US1	GCA_026123055.1
Ca. Electrothrix communis US2	GCA_026123035.1
Ca. Electrothrix communis US4	GCA_026123015.1
Ca. Electrothrix communis US5	GCA_026122995.1
Ca. Electrothrix EH2	GCA_022765845.1
Ca. Electrothrix gigas AS–4_5	GCA_022765825.1
Ca. Electrothrix gigas AU–1_5	GCA_022766165.1
Ca. Electrothrix gigas AUS–3	GCA_022765875.1
Ca. Electrothrix gigas AW–1	GCA_022765985.1
Ca. Electrothrix gigas AW–2	GCA_022765945.1
Ca. Electrothrix gigas AW–3_4	GCA_022766005.1
Ca. Electrothrix gigas AW–5	GCA_022766025.1
Ca. Electrothrix gigas AX–1_4	GCA_022766045.1

**Species**	**Genome accession**
Ca. Electrothrix gigas AX–2	GCA_022766065.1
Ca. Electrothrix gigas HOU-hqMAG	GCA_942491745.1
Ca. Electrothrix gigas LOE–1_4_5	GCA_022766095.1
Ca. Electrothrix GM3_4	GCA_022765805.1
Ca. Electrothrix japonica TB	GCA_026122855.1
Ca. Electrothrix laxa	GCA_942492895.1
Ca. Electrothrix LOE2	GCA_022766145.1
Ca. Electrothrix MAN1_4	GCA_022766125.1
Ca. Electrothrix marina A3	GCA_004028525.1
Ca. Electrothrix marina A2	GCA_004028515.1
Ca. Electrothrix scaldis GW3-3	GCA_033584155.1
Ca. Electrothrix SY2	GCA_011389815.1
Unclassified *Desulfobulbaceae* AR-3	GCA_022765765.1
Unclassified *Desulfobulbaceae* AR-4	GCA_022765725.1
Desulfobulbus alkaliphus	GCF_016918545.1
Desulfobulbus elongatus	GCF_000621145.1
Desulfobulbus oligotrophicus	GCF_016446285.1
Desulfobulbus oralis	GCF_002952055.1
Desulfobulbus propionicus	GCF_000186885.1
Desulfobulbus rhabdoformis	GCF_016919065.1
Desulfocapsa sulfexigens	GCF_000341395.1
Desulfofustis glycolicus	GCF_900130015.1
Desulfogranum japonicum	GCF_000429945.1
Desulfogranum marinum	GCF_016918565.1
Desulfogranum mediterraneum	GCF_000429965.1
Desulfolithobacter dissulfuricans	GCF_025998535.1
Desulfomarina profundi	GCF_019703855.1
Desulfopila aestuarrii	GCF_900143695.1
Desulfoprunum benzoelyticum	GCF_014201505.1
Desulforhopalus singaporensis	GCF_900104445.1
Desulforhopalus vacuolatus	GCF_016918505.1
Desulfotalea psychrophila	GCF_000025945.1
Desulfurivibrio alkaliphus	GCF_000092205.1
